# Measurement of the centrality dependence of the charged-particle pseudorapidity distribution in proton–lead collisions at $\sqrt{s_{_\text {NN}}} = 5.02$ TeV with the ATLAS detector

**DOI:** 10.1140/epjc/s10052-016-4002-3

**Published:** 2016-04-12

**Authors:** G. Aad, T. Abajyan, B. Abbott, J. Abdallah, S. Abdel Khalek, O. Abdinov, R. Aben, B. Abi, M. Abolins, O. S. AbouZeid, H. Abramowicz, H. Abreu, Y. Abulaiti, B. S. Acharya, L. Adamczyk, D. L. Adams, T. N. Addy, J. Adelman, S. Adomeit, T. Adye, T. Agatonovic-Jovin, J. A. Aguilar-Saavedra, M. Agustoni, S. P. Ahlen, F. Ahmadov, G. Aielli, T. P. A. Åkesson, G. Akimoto, A. V. Akimov, J. Albert, S. Albrand, M. J. Alconada Verzini, M. Aleksa, I. N. Aleksandrov, C. Alexa, G. Alexander, G. Alexandre, T. Alexopoulos, M. Alhroob, G. Alimonti, L. Alio, J. Alison, B. M. M. Allbrooke, L. J. Allison, P. P. Allport, S. E. Allwood-Spiers, J. Almond, A. Aloisio, R. Alon, A. Alonso, F. Alonso, C. Alpigiani, A. Altheimer, B. Alvarez Gonzalez, M. G. Alviggi, K. Amako, Y. Amaral Coutinho, C. Amelung, V. V. Ammosov, S. P. Amor Dos Santos, A. Amorim, S. Amoroso, N. Amram, G. Amundsen, C. Anastopoulos, L. S. Ancu, N. Andari, T. Andeen, C. F. Anders, G. Anders, K. J. Anderson, A. Andreazza, V. Andrei, X. S. Anduaga, S. Angelidakis, P. Anger, A. Angerami, F. Anghinolfi, A. V. Anisenkov, N. Anjos, A. Annovi, A. Antonaki, M. Antonelli, A. Antonov, J. Antos, F. Anulli, M. Aoki, L. Aperio Bella, R. Apolle, G. Arabidze, I. Aracena, Y. Arai, A. T. H. Arce, J-F. Arguin, S. Argyropoulos, M. Arik, A. J. Armbruster, O. Arnaez, V. Arnal, O. Arslan, A. Artamonov, G. Artoni, S. Asai, N. Asbah, S. Ask, B. Åsman, L. Asquith, K. Assamagan, R. Astalos, M. Atkinson, N. B. Atlay, B. Auerbach, E. Auge, K. Augsten, M. Aurousseau, G. Avolio, G. Azuelos, Y. Azuma, M. A. Baak, C. Bacci, A. M. Bach, H. Bachacou, K. Bachas, M. Backes, M. Backhaus, J. Backus Mayes, E. Badescu, P. Bagiacchi, P. Bagnaia, Y. Bai, D. C. Bailey, T. Bain, J. T. Baines, O. K. Baker, S. Baker, P. Balek, F. Balli, E. Banas, Sw. Banerjee, A. Bangert, V. Bansal, H. S. Bansil, L. Barak, T. Barber, E. L. Barberio, D. Barberis, M. Barbero, T. Barillari, M. Barisonzi, T. Barklow, N. Barlow, B. M. Barnett, R. M. Barnett, A. Baroncelli, G. Barone, A. J. Barr, F. Barreiro, J. Barreiro Guimarães da Costa, R. Bartoldus, A. E. Barton, P. Bartos, V. Bartsch, A. Bassalat, A. Basye, R. L. Bates, L. Batkova, J. R. Batley, M. Battistin, F. Bauer, H. S. Bawa, T. Beau, P. H. Beauchemin, R. Beccherle, P. Bechtle, H. P. Beck, K. Becker, S. Becker, M. Beckingham, A. J. Beddall, A. Beddall, S. Bedikian, V. A. Bednyakov, C. P. Bee, L. J. Beemster, T. A. Beermann, M. Begel, J. K. Behr, C. Belanger-Champagne, P. J. Bell, W. H. Bell, G. Bella, L. Bellagamba, A. Bellerive, M. Bellomo, A. Belloni, K. Belotskiy, O. Beltramello, O. Benary, D. Benchekroun, K. Bendtz, N. Benekos, Y. Benhammou, E. Benhar Noccioli, J. A. Benitez Garcia, D. P. Benjamin, J. R. Bensinger, K. Benslama, S. Bentvelsen, D. Berge, E. Bergeaas Kuutmann, N. Berger, F. Berghaus, E. Berglund, J. Beringer, C. Bernard, P. Bernat, C. Bernius, F. U. Bernlochner, T. Berry, P. Berta, C. Bertella, F. Bertolucci, M. I. Besana, G. J. Besjes, O. Bessidskaia Bylund, N. Besson, C. Betancourt, S. Bethke, W. Bhimji, R. M. Bianchi, L. Bianchini, M. Bianco, O. Biebel, S. P. Bieniek, K. Bierwagen, J. Biesiada, M. Biglietti, J. Bilbao De Mendizabal, H. Bilokon, M. Bindi, S. Binet, A. Bingul, C. Bini, C. W. Black, J. E. Black, K. M. Black, D. Blackburn, R. E. Blair, J.-B. Blanchard, T. Blazek, I. Bloch, C. Blocker, W. Blum, U. Blumenschein, G. J. Bobbink, V. S. Bobrovnikov, S. S. Bocchetta, A. Bocci, C. R. Boddy, M. Boehler, J. Boek, T. T. Boek, J. A. Bogaerts, A. G. Bogdanchikov, A. Bogouch, C. Bohm, J. Bohm, V. Boisvert, T. Bold, V. Boldea, A. S. Boldyrev, N. M. Bolnet, M. Bomben, M. Bona, M. Boonekamp, A. Borisov, G. Borissov, M. Borri, S. Borroni, J. Bortfeldt, V. Bortolotto, K. Bos, D. Boscherini, M. Bosman, H. Boterenbrood, J. Boudreau, J. Bouffard, E. V. Bouhova-Thacker, D. Boumediene, C. Bourdarios, N. Bousson, S. Boutouil, A. Boveia, J. Boyd, I. R. Boyko, I. Bozovic-Jelisavcic, J. Bracinik, P. Branchini, A. Brandt, G. Brandt, O. Brandt, U. Bratzler, B. Brau, J. E. Brau, H. M. Braun, S. F. Brazzale, B. Brelier, K. Brendlinger, A. J. Brennan, R. Brenner, S. Bressler, K. Bristow, T. M. Bristow, D. Britton, F. M. Brochu, I. Brock, R. Brock, C. Bromberg, J. Bronner, G. Brooijmans, T. Brooks, W. K. Brooks, J. Brosamer, E. Brost, G. Brown, J. Brown, P. A. Bruckman de Renstrom, D. Bruncko, R. Bruneliere, S. Brunet, A. Bruni, G. Bruni, M. Bruschi, L. Bryngemark, T. Buanes, Q. Buat, F. Bucci, P. Buchholz, R. M. Buckingham, A. G. Buckley, S. I. Buda, I. A. Budagov, F. Buehrer, L. Bugge, M. K. Bugge, O. Bulekov, A. C. Bundock, H. Burckhart, S. Burdin, B. Burghgrave, S. Burke, I. Burmeister, E. Busato, V. Büscher, P. Bussey, C. P. Buszello, B. Butler, J. M. Butler, A. I. Butt, C. M. Buttar, J. M. Butterworth, W. Buttinger, A. Buzatu, M. Byszewski, S. Cabrera Urbán, D. Caforio, O. Cakir, P. Calafiura, G. Calderini, P. Calfayan, R. Calkins, L. P. Caloba, D. Calvet, S. Calvet, R. Camacho Toro, D. Cameron, L. M. Caminada, R. Caminal Armadans, S. Campana, M. Campanelli, A. Campoverde, V. Canale, A. Canepa, J. Cantero, R. Cantrill, T. Cao, M. D. M. Capeans Garrido, I. Caprini, M. Caprini, M. Capua, R. Caputo, R. Cardarelli, T. Carli, G. Carlino, L. Carminati, S. Caron, E. Carquin, G. D. Carrillo-Montoya, J. R. Carter, J. Carvalho, D. Casadei, M. P. Casado, E. Castaneda-Miranda, A. Castelli, V. Castillo Gimenez, N. F. Castro, P. Catastini, A. Catinaccio, J. R. Catmore, A. Cattai, G. Cattani, S. Caughron, V. Cavaliere, D. Cavalli, M. Cavalli-Sforza, V. Cavasinni, F. Ceradini, B. C. Cerio, K. Cerny, A. S. Cerqueira, A. Cerri, L. Cerrito, F. Cerutti, M. Cerv, A. Cervelli, S. A. Cetin, A. Chafaq, D. Chakraborty, I. Chalupkova, K. Chan, P. Chang, B. Chapleau, J. D. Chapman, D. Charfeddine, D. G. Charlton, C. A. Chavez Barajas, S. Cheatham, S. Chekanov, S. V. Chekulaev, G. A. Chelkov, M. A. Chelstowska, C. Chen, H. Chen, K. Chen, L. Chen, S. Chen, X. Chen, Y. Chen, H. C. Cheng, Y. Cheng, A. Cheplakov, R. Cherkaoui El Moursli, V. Chernyatin, E. Cheu, L. Chevalier, V. Chiarella, G. Chiefari, J. T. Childers, A. Chilingarov, G. Chiodini, A. S. Chisholm, R. T. Chislett, A. Chitan, M. V. Chizhov, S. Chouridou, B. K. B. Chow, I. A. Christidi, D. Chromek-Burckhart, M. L. Chu, J. Chudoba, L. Chytka, G. Ciapetti, A. K. Ciftci, R. Ciftci, D. Cinca, V. Cindro, A. Ciocio, P. Cirkovic, Z. H. Citron, M. Ciubancan, A. Clark, P. J. Clark, R. N. Clarke, W. Cleland, J. C. Clemens, B. Clement, C. Clement, Y. Coadou, M. Cobal, A. Coccaro, J. Cochran, L. Coffey, J. G. Cogan, J. Coggeshall, B. Cole, S. Cole, A. P. Colijn, C. Collins-Tooth, J. Collot, T. Colombo, G. Colon, G. Compostella, P. Conde Muiño, E. Coniavitis, M. C. Conidi, I. A. Connelly, S. M. Consonni, V. Consorti, S. Constantinescu, C. Conta, G. Conti, F. Conventi, M. Cooke, B. D. Cooper, A. M. Cooper-Sarkar, N. J. Cooper-Smith, K. Copic, T. Cornelissen, M. Corradi, F. Corriveau, A. Corso-Radu, A. Cortes-Gonzalez, G. Cortiana, G. Costa, M. J. Costa, D. Costanzo, D. Côté, G. Cottin, G. Cowan, B. E. Cox, K. Cranmer, G. Cree, S. Crépé-Renaudin, F. Crescioli, M. Crispin Ortuzar, M. Cristinziani, G. Crosetti, C.-M. Cuciuc, T. Cuhadar Donszelmann, J. Cummings, M. Curatolo, C. Cuthbert, H. Czirr, P. Czodrowski, Z. Czyczula, S. D’Auria, M. D’Onofrio, M. J. Da Cunha Sargedas De Sousa, C. Da Via, W. Dabrowski, A. Dafinca, T. Dai, O. Dale, F. Dallaire, C. Dallapiccola, M. Dam, A. C. Daniells, M. Dano Hoffmann, V. Dao, G. Darbo, G. L. Darlea, S. Darmora, J. Dassoulas, W. Davey, C. David, T. Davidek, E. Davies, M. Davies, O. Davignon, A. R. Davison, P. Davison, Y. Davygora, E. Dawe, I. Dawson, R. K. Daya-Ishmukhametova, K. De, R. de Asmundis, S. De Castro, S. De Cecco, J. de Graat, N. De Groot, P. de Jong, C. De La Taille, H. De la Torre, F. De Lorenzi, L. De Nooij, D. De Pedis, A. De Salvo, U. De Sanctis, A. De Santo, J. B. De Vivie De Regie, G. De Zorzi, W. J. Dearnaley, R. Debbe, C. Debenedetti, B. Dechenaux, D. V. Dedovich, J. Degenhardt, I. Deigaard, J. Del Peso, T. Del Prete, T. Delemontex, F. Deliot, M. Deliyergiyev, A. Dell’Acqua, L. Dell’Asta, M. Della Pietra, D. della Volpe, M. Delmastro, P. A. Delsart, C. Deluca, S. Demers, M. Demichev, A. Demilly, S. P. Denisov, D. Derendarz, J. E. Derkaoui, F. Derue, P. Dervan, K. Desch, C. Deterre, P. O. Deviveiros, A. Dewhurst, S. Dhaliwal, A. Di Ciaccio, L. Di Ciaccio, A. Di Domenico, C. Di Donato, A. Di Girolamo, B. Di Girolamo, A. Di Mattia, B. Di Micco, R. Di Nardo, A. Di Simone, R. Di Sipio, D. Di Valentino, M. A. Diaz, E. B. Diehl, J. Dietrich, T. A. Dietzsch, S. Diglio, A. Dimitrievska, J. Dingfelder, C. Dionisi, P. Dita, S. Dita, F. Dittus, F. Djama, T. Djobava, J. I. Djuvsland, M. A. B. do Vale, A. Do Valle Wemans, T. K. O. Doan, D. Dobos, E. Dobson, C. Doglioni, T. Doherty, T. Dohmae, J. Dolejsi, Z. Dolezal, B. A. Dolgoshein, M. Donadelli, S. Donati, P. Dondero, J. Donini, J. Dopke, A. Doria, A. Dotti, M. T. Dova, A. T. Doyle, M. Dris, J. Dubbert, S. Dube, E. Dubreuil, E. Duchovni, G. Duckeck, O. A. Ducu, D. Duda, A. Dudarev, F. Dudziak, L. Duflot, L. Duguid, M. Dührssen, M. Dunford, H. Duran Yildiz, M. Düren, M. Dwuznik, J. Ebke, W. Edson, N. C. Edwards, W. Ehrenfeld, T. Eifert, G. Eigen, K. Einsweiler, T. Ekelof, M. El Kacimi, M. Ellert, S. Elles, F. Ellinghaus, N. Ellis, J. Elmsheuser, M. Elsing, D. Emeliyanov, Y. Enari, O. C. Endner, M. Endo, J. Erdmann, A. Ereditato, D. Eriksson, G. Ernis, J. Ernst, M. Ernst, J. Ernwein, D. Errede, S. Errede, E. Ertel, M. Escalier, H. Esch, C. Escobar, X. Espinal Curull, B. Esposito, A. I. Etienvre, E. Etzion, H. Evans, L. Fabbri, G. Facini, R. M. Fakhrutdinov, S. Falciano, J. Faltova, Y. Fang, M. Fanti, A. Farbin, A. Farilla, T. Farooque, S. Farrell, S. M. Farrington, P. Farthouat, F. Fassi, P. Fassnacht, D. Fassouliotis, A. Favareto, L. Fayard, P. Federic, O. L. Fedin, W. Fedorko, M. Fehling-Kaschek, S. Feigl, L. Feligioni, C. Feng, E. J. Feng, H. Feng, A. B. Fenyuk, S. Fernandez Perez, W. Fernando, S. Ferrag, J. Ferrando, V. Ferrara, A. Ferrari, P. Ferrari, R. Ferrari, D. E. Ferreira de Lima, A. Ferrer, D. Ferrere, C. Ferretti, A. Ferretto Parodi, M. Fiascaris, F. Fiedler, A. Filipčič, M. Filipuzzi, F. Filthaut, M. Fincke-Keeler, K. D. Finelli, M. C. N. Fiolhais, L. Fiorini, A. Firan, J. Fischer, M. J. Fisher, E. A. Fitzgerald, M. Flechl, I. Fleck, P. Fleischmann, S. Fleischmann, G. T. Fletcher, G. Fletcher, T. Flick, A. Floderus, L. R. Flores Castillo, A. C. Florez Bustos, M. J. Flowerdew, A. Formica, A. Forti, D. Fortin, D. Fournier, H. Fox, P. Francavilla, M. Franchini, S. Franchino, D. Francis, M. Franklin, S. Franz, M. Fraternali, S. Fratina, S. T. French, C. Friedrich, F. Friedrich, D. Froidevaux, J. A. Frost, C. Fukunaga, E. Fullana Torregrosa, B. G. Fulsom, J. Fuster, C. Gabaldon, O. Gabizon, A. Gabrielli, A. Gabrielli, S. Gadatsch, S. Gadomski, G. Gagliardi, P. Gagnon, C. Galea, B. Galhardo, E. J. Gallas, V. Gallo, B. J. Gallop, P. Gallus, G. Galster, K. K. Gan, R. P. Gandrajula, J. Gao, Y. S. Gao, F. M. Garay Walls, F. Garberson, C. García, J. E. García Navarro, M. Garcia-Sciveres, R. W. Gardner, N. Garelli, V. Garonne, C. Gatti, G. Gaudio, B. Gaur, L. Gauthier, P. Gauzzi, I. L. Gavrilenko, C. Gay, G. Gaycken, E. N. Gazis, P. Ge, Z. Gecse, C. N. P. Gee, D. A. A. Geerts, Ch. Geich-Gimbel, C. Gemme, A. Gemmell, M. H. Genest, S. Gentile, M. George, S. George, D. Gerbaudo, A. Gershon, H. Ghazlane, N. Ghodbane, B. Giacobbe, S. Giagu, V. Giangiobbe, P. Giannetti, F. Gianotti, B. Gibbard, S. M. Gibson, M. Gilchriese, T. P. S. Gillam, D. Gillberg, A. R. Gillman, D. M. Gingrich, N. Giokaris, M. P. Giordani, R. Giordano, F. M. Giorgi, P. F. Giraud, D. Giugni, C. Giuliani, M. Giunta, B. K. Gjelsten, I. Gkialas, L. K. Gladilin, C. Glasman, J. Glatzer, A. Glazov, G. L. Glonti, M. Goblirsch-Kolb, J. R. Goddard, J. Godfrey, J. Godlewski, C. Goeringer, S. Goldfarb, T. Golling, D. Golubkov, A. Gomes, L. S. Gomez Fajardo, R. Gonçalo, J. Goncalves Pinto Firmino Da Costa, L. Gonella, S. González de la Hoz, G. Gonzalez Parra, M. L. Gonzalez Silva, S. Gonzalez-Sevilla, L. Goossens, P. A. Gorbounov, H. A. Gordon, I. Gorelov, B. Gorini, E. Gorini, A. Gorišek, E. Gornicki, A. T. Goshaw, C. Gössling, M. I. Gostkin, M. Gouighri, D. Goujdami, M. P. Goulette, A. G. Goussiou, C. Goy, S. Gozpinar, H. M. X. Grabas, L. Graber, I. Grabowska-Bold, P. Grafström, K-J. Grahn, J. Gramling, E. Gramstad, F. Grancagnolo, S. Grancagnolo, V. Grassi, V. Gratchev, H. M. Gray, E. Graziani, O. G. Grebenyuk, Z. D. Greenwood, K. Gregersen, I. M. Gregor, P. Grenier, J. Griffiths, A. A. Grillo, K. Grimm, S. Grinstein, Ph. Gris, Y. V. Grishkevich, J.-F. Grivaz, J. P. Grohs, A. Grohsjean, E. Gross, J. Grosse-Knetter, G. C. Grossi, J. Groth-Jensen, Z. J. Grout, K. Grybel, L. Guan, J. Guenther, F. Guescini, D. Guest, O. Gueta, C. Guicheney, E. Guido, T. Guillemin, S. Guindon, U. Gul, C. Gumpert, J. Guo, S. Gupta, P. Gutierrez, N. G. Gutierrez Ortiz, C. Gutschow, N. Guttman, C. Guyot, C. Gwenlan, C. B. Gwilliam, A. Haas, C. Haber, H. K. Hadavand, N. Haddad, P. Haefner, S. Hageböck, Z. Hajduk, H. Hakobyan, M. Haleem, D. Hall, G. Halladjian, K. Hamacher, P. Hamal, K. Hamano, M. Hamer, A. Hamilton, S. Hamilton, L. Han, K. Hanagaki, K. Hanawa, M. Hance, P. Hanke, J. B. Hansen, J. D. Hansen, P. H. Hansen, K. Hara, A. S. Hard, T. Harenberg, S. Harkusha, D. Harper, R. D. Harrington, O. M. Harris, P. F. Harrison, F. Hartjes, A. Harvey, S. Hasegawa, Y. Hasegawa, S. Hassani, S. Haug, M. Hauschild, R. Hauser, M. Havranek, C. M. Hawkes, R. J. Hawkings, A. D. Hawkins, T. Hayashi, D. Hayden, C. P. Hays, H. S. Hayward, S. J. Haywood, S. J. Head, T. Heck, V. Hedberg, L. Heelan, S. Heim, T. Heim, B. Heinemann, L. Heinrich, S. Heisterkamp, J. Hejbal, L. Helary, C. Heller, M. Heller, S. Hellman, D. Hellmich, C. Helsens, J. Henderson, R. C. W. Henderson, C. Hengler, A. Henrichs, A. M. Henriques Correia, S. Henrot-Versille, C. Hensel, G. H. Herbert, Y. Hernández Jiménez, R. Herrberg-Schubert, G. Herten, R. Hertenberger, L. Hervas, G. G. Hesketh, N. P. Hessey, R. Hickling, E. Higón-Rodriguez, J. C. Hill, K. H. Hiller, S. Hillert, S. J. Hillier, I. Hinchliffe, E. Hines, M. Hirose, D. Hirschbuehl, J. Hobbs, N. Hod, M. C. Hodgkinson, P. Hodgson, A. Hoecker, M. R. Hoeferkamp, J. Hoffman, D. Hoffmann, M. Hohlfeld, T. R. Holmes, T. M. Hong, L. Hooft van Huysduynen, J-Y. Hostachy, S. Hou, A. Hoummada, J. Howard, J. Howarth, M. Hrabovsky, I. Hristova, J. Hrivnac, T. Hryn’ova, P. J. Hsu, S.-C. Hsu, D. Hu, X. Hu, Y. Huang, Z. Hubacek, F. Hubaut, F. Huegging, T. B. Huffman, E. W. Hughes, G. Hughes, M. Huhtinen, T. A. Hülsing, M. Hurwitz, N. Huseynov, J. Huston, J. Huth, G. Iacobucci, G. Iakovidis, I. Ibragimov, L. Iconomidou-Fayard, E. Ideal, P. Iengo, O. Igonkina, T. Iizawa, Y. Ikegami, K. Ikematsu, M. Ikeno, D. Iliadis, N. Ilic, Y. Inamaru, T. Ince, P. Ioannou, M. Iodice, K. Iordanidou, V. Ippolito, A. Irles Quiles, C. Isaksson, M. Ishino, M. Ishitsuka, R. Ishmukhametov, C. Issever, S. Istin, J. M. Iturbe Ponce, A. V. Ivashin, W. Iwanski, H. Iwasaki, J. M. Izen, V. Izzo, B. Jackson, J. N. Jackson, M. Jackson, P. Jackson, M. R. Jaekel, V. Jain, K. Jakobs, S. Jakobsen, T. Jakoubek, J. Jakubek, D. O. Jamin, D. K. Jana, E. Jansen, H. Jansen, J. Janssen, M. Janus, G. Jarlskog, L. Jeanty, G.-Y. Jeng, I. Jen-La Plante, D. Jennens, P. Jenni, J. Jentzsch, C. Jeske, S. Jézéquel, H. Ji, W. Ji, J. Jia, Y. Jiang, M. Jimenez Belenguer, S. Jin, A. Jinaru, O. Jinnouchi, M. D. Joergensen, D. Joffe, K. E. Johansson, P. Johansson, K. A. Johns, K. Jon-And, G. Jones, R. W. L. Jones, T. J. Jones, P. M. Jorge, K. D. Joshi, J. Jovicevic, X. Ju, C. A. Jung, R. M. Jungst, P. Jussel, A. Juste Rozas, M. Kaci, A. Kaczmarska, M. Kado, H. Kagan, M. Kagan, E. Kajomovitz, S. Kama, N. Kanaya, M. Kaneda, S. Kaneti, T. Kanno, V. A. Kantserov, J. Kanzaki, B. Kaplan, A. Kapliy, D. Kar, K. Karakostas, N. Karastathis, M. Karnevskiy, S. N. Karpov, K. Karthik, V. Kartvelishvili, A. N. Karyukhin, L. Kashif, G. Kasieczka, R. D. Kass, A. Kastanas, Y. Kataoka, A. Katre, J. Katzy, V. Kaushik, K. Kawagoe, T. Kawamoto, G. Kawamura, S. Kazama, V. F. Kazanin, M. Y. Kazarinov, R. Keeler, R. Kehoe, M. Keil, J. S. Keller, H. Keoshkerian, O. Kepka, B. P. Kerševan, S. Kersten, K. Kessoku, J. Keung, F. Khalil-zada, H. Khandanyan, A. Khanov, A. Khodinov, A. Khomich, T. J. Khoo, G. Khoriauli, A. Khoroshilov, V. Khovanskiy, E. Khramov, J. Khubua, H. Kim, S. H. Kim, N. Kimura, O. M. Kind, B. T. King, M. King, R. S. B. King, S. B. King, J. Kirk, A. E. Kiryunin, T. Kishimoto, D. Kisielewska, F. Kiss, T. Kitamura, T. Kittelmann, K. Kiuchi, E. Kladiva, M. Klein, U. Klein, K. Kleinknecht, P. Klimek, A. Klimentov, R. Klingenberg, J. A. Klinger, E. B. Klinkby, T. Klioutchnikova, P. F. Klok, E.-E. Kluge, P. Kluit, S. Kluth, E. Kneringer, E. B. F. G. Knoops, A. Knue, T. Kobayashi, M. Kobel, M. Kocian, P. Kodys, P. Koevesarki, T. Koffas, E. Koffeman, L. A. Kogan, S. Kohlmann, Z. Kohout, T. Kohriki, T. Koi, H. Kolanoski, I. Koletsou, J. Koll, A. A. Komar, Y. Komori, T. Kondo, K. Köneke, A. C. König, S. König, T. Kono, R. Konoplich, N. Konstantinidis, R. Kopeliansky, S. Koperny, L. Köpke, A. K. Kopp, K. Korcyl, K. Kordas, A. Korn, A. A. Korol, I. Korolkov, E. V. Korolkova, V. A. Korotkov, O. Kortner, S. Kortner, V. V. Kostyukhin, V. M. Kotov, A. Kotwal, C. Kourkoumelis, V. Kouskoura, A. Koutsman, R. Kowalewski, T. Z. Kowalski, W. Kozanecki, A. S. Kozhin, V. Kral, V. A. Kramarenko, G. Kramberger, D. Krasnopevtsev, M. W. Krasny, A. Krasznahorkay, J. K. Kraus, A. Kravchenko, S. Kreiss, M. Kretz, J. Kretzschmar, K. Kreutzfeldt, P. Krieger, K. Kroeninger, H. Kroha, J. Kroll, J. Kroseberg, J. Krstic, U. Kruchonak, H. Krüger, T. Kruker, N. Krumnack, Z. V. Krumshteyn, A. Kruse, M. C. Kruse, M. Kruskal, T. Kubota, S. Kuday, S. Kuehn, A. Kugel, A. Kuhl, T. Kuhl, V. Kukhtin, Y. Kulchitsky, S. Kuleshov, M. Kuna, J. Kunkle, A. Kupco, H. Kurashige, Y. A. Kurochkin, R. Kurumida, V. Kus, E. S. Kuwertz, M. Kuze, J. Kvita, A. La Rosa, L. La Rotonda, L. Labarga, C. Lacasta, F. Lacava, J. Lacey, H. Lacker, D. Lacour, V. R. Lacuesta, E. Ladygin, R. Lafaye, B. Laforge, T. Lagouri, S. Lai, H. Laier, E. Laisne, L. Lambourne, C. L. Lampen, W. Lampl, E. Lançon, U. Landgraf, M. P. J. Landon, V. S. Lang, C. Lange, A. J. Lankford, F. Lanni, K. Lantzsch, S. Laplace, C. Lapoire, J. F. Laporte, T. Lari, M. Lassnig, P. Laurelli, V. Lavorini, W. Lavrijsen, P. Laycock, B. T. Le, O. Le Dortz, E. Le Guirriec, E. Le Menedeu, T. LeCompte, F. Ledroit-Guillon, C. A. Lee, H. Lee, J. S. H. Lee, S. C. Lee, L. Lee, G. Lefebvre, M. Lefebvre, F. Legger, C. Leggett, A. Lehan, M. Lehmacher, G. Lehmann Miotto, X. Lei, A. G. Leister, M. A. L. Leite, R. Leitner, D. Lellouch, B. Lemmer, K. J. C. Leney, T. Lenz, B. Lenzi, R. Leone, K. Leonhardt, S. Leontsinis, C. Leroy, C. G. Lester, C. M. Lester, J. Levêque, D. Levin, L. J. Levinson, A. Lewis, G. H. Lewis, A. M. Leyko, M. Leyton, B. Li, B. Li, H. Li, H. L. Li, S. Li, X. Li, Z. Liang, H. Liao, B. Liberti, P. Lichard, K. Lie, J. Liebal, W. Liebig, C. Limbach, A. Limosani, M. Limper, S. C. Lin, F. Linde, B. E. Lindquist, J. T. Linnemann, E. Lipeles, A. Lipniacka, M. Lisovyi, T. M. Liss, D. Lissauer, A. Lister, A. M. Litke, B. Liu, D. Liu, J. B. Liu, K. Liu, L. Liu, M. Liu, M. Liu, Y. Liu, M. Livan, S. S. A. Livermore, A. Lleres, J. Llorente Merino, S. L. Lloyd, F. Lo Sterzo, E. Lobodzinska, P. Loch, W. S. Lockman, F. K. Loebinger, A. E. Loevschall-Jensen, A. Loginov, C. W. Loh, T. Lohse, K. Lohwasser, M. Lokajicek, V. P. Lombardo, J. D. Long, R. E. Long, L. Lopes, D. Lopez Mateos, B. Lopez Paredes, J. Lorenz, N. Lorenzo Martinez, M. Losada, P. Loscutoff, M. J. Losty, X. Lou, A. Lounis, J. Love, P. A. Love, A. J. Lowe, H. J. Lubatti, C. Luci, A. Lucotte, D. Ludwig, F. Luehring, W. Lukas, L. Luminari, O. Lundberg, B. Lund-Jensen, M. Lungwitz, D. Lynn, R. Lysak, E. Lytken, H. Ma, L. L. Ma, G. Maccarrone, A. Macchiolo, J. Machado Miguens, D. Macina, R. Mackeprang, R. Madar, H. J. Maddocks, W. F. Mader, A. Madsen, T. Maeno, M. Maeno Kataoka, E. Magradze, K. Mahboubi, J. Mahlstedt, S. Mahmoud, C. Maiani, C. Maidantchik, A. Maio, S. Majewski, Y. Makida, N. Makovec, P. Mal, B. Malaescu, Pa. Malecki, V. P. Maleev, F. Malek, U. Mallik, D. Malon, C. Malone, S. Maltezos, V. M. Malyshev, S. Malyukov, J. Mamuzic, B. Mandelli, L. Mandelli, I. Mandić, R. Mandrysch, J. Maneira, A. Manfredini, L. Manhaes de Andrade Filho, J. Manjarres Ramos, A. Mann, P. M. Manning, A. Manousakis-Katsikakis, B. Mansoulie, R. Mantifel, L. Mapelli, L. March, J. F. Marchand, F. Marchese, G. Marchiori, M. Marcisovsky, C. P. Marino, C. N. Marques, F. Marroquim, S. P. Marsden, Z. Marshall, L. F. Marti, S. Marti-Garcia, B. Martin, B. Martin, T. A. Martin, V. J. Martin, B. Martin dit Latour, H. Martinez, M. Martinez, S. Martin-Haugh, A. C. Martyniuk, M. Marx, F. Marzano, A. Marzin, L. Masetti, T. Mashimo, R. Mashinistov, J. Masik, A. L. Maslennikov, I. Massa, N. Massol, P. Mastrandrea, A. Mastroberardino, T. Masubuchi, H. Matsunaga, T. Matsushita, P. Mättig, S. Mättig, J. Mattmann, J. Maurer, S. J. Maxfield, D. A. Maximov, R. Mazini, L. Mazzaferro, G. Mc Goldrick, S. P. Mc Kee, A. McCarn, R. L. McCarthy, T. G. McCarthy, N. A. McCubbin, K. W. McFarlane, J. A. Mcfayden, G. Mchedlidze, T. Mclaughlan, S. J. McMahon, R. A. McPherson, A. Meade, J. Mechnich, M. Mechtel, M. Medinnis, S. Meehan, R. Meera-Lebbai, S. Mehlhase, A. Mehta, K. Meier, C. Meineck, B. Meirose, C. Melachrinos, B. R. Mellado Garcia, F. Meloni, L. Mendoza Navas, A. Mengarelli, S. Menke, E. Meoni, K. M. Mercurio, S. Mergelmeyer, N. Meric, P. Mermod, L. Merola, C. Meroni, F. S. Merritt, H. Merritt, A. Messina, J. Metcalfe, A. S. Mete, C. Meyer, C. Meyer, J-P. Meyer, J. Meyer, R. P. Middleton, S. Migas, L. Mijović, G. Mikenberg, M. Mikestikova, M. Mikuž, D. W. Miller, C. Mills, A. Milov, D. A. Milstead, D. Milstein, A. A. Minaenko, M. Miñano Moya, I. A. Minashvili, A. I. Mincer, B. Mindur, M. Mineev, Y. Ming, L. M. Mir, G. Mirabelli, T. Mitani, J. Mitrevski, V. A. Mitsou, S. Mitsui, A. Miucci, P. S. Miyagawa, J. U. Mjörnmark, T. Moa, V. Moeller, S. Mohapatra, W. Mohr, S. Molander, R. Moles-Valls, K. Mönig, C. Monini, J. Monk, E. Monnier, J. Montejo Berlingen, F. Monticelli, S. Monzani, R. W. Moore, C. Mora Herrera, A. Moraes, N. Morange, J. Morel, D. Moreno, M. Moreno Llácer, P. Morettini, M. Morgenstern, M. Morii, S. Moritz, A. K. Morley, G. Mornacchi, J. D. Morris, L. Morvaj, H. G. Moser, M. Mosidze, J. Moss, R. Mount, E. Mountricha, S. V. Mouraviev, E. J. W. Moyse, S. Muanza, R. D. Mudd, F. Mueller, J. Mueller, K. Mueller, T. Mueller, T. Mueller, D. Muenstermann, Y. Munwes, J. A. Murillo Quijada, W. J. Murray, E. Musto, A. G. Myagkov, M. Myska, O. Nackenhorst, J. Nadal, K. Nagai, R. Nagai, Y. Nagai, K. Nagano, A. Nagarkar, Y. Nagasaka, M. Nagel, A. M. Nairz, Y. Nakahama, K. Nakamura, T. Nakamura, I. Nakano, H. Namasivayam, G. Nanava, R. Narayan, T. Nattermann, T. Naumann, G. Navarro, R. Nayyar, H. A. Neal, P. Yu. Nechaeva, T. J. Neep, A. Negri, G. Negri, M. Negrini, S. Nektarijevic, A. Nelson, T. K. Nelson, S. Nemecek, P. Nemethy, A. A. Nepomuceno, M. Nessi, M. S. Neubauer, M. Neumann, A. Neusiedl, R. M. Neves, P. Nevski, P. R. Newman, D. H. Nguyen, R. B. Nickerson, R. Nicolaidou, B. Nicquevert, J. Nielsen, N. Nikiforou, A. Nikiforov, V. Nikolaenko, I. Nikolic-Audit, K. Nikolics, K. Nikolopoulos, P. Nilsson, Y. Ninomiya, A. Nisati, R. Nisius, T. Nobe, L. Nodulman, M. Nomachi, I. Nomidis, S. Norberg, M. Nordberg, S. Nowak, M. Nozaki, L. Nozka, K. Ntekas, A.-E. Nuncio-Quiroz, G. Nunes Hanninger, T. Nunnemann, E. Nurse, F. Nuti, B. J. O’Brien, F. O’grady, D. C. O’Neil, V. O’Shea, F. G. Oakham, H. Oberlack, J. Ocariz, A. Ochi, I. Ochoa, S. Oda, S. Odaka, H. Ogren, A. Oh, S. H. Oh, C. C. Ohm, H. Ohman, T. Ohshima, W. Okamura, H. Okawa, Y. Okumura, T. Okuyama, A. Olariu, A. G. Olchevski, S. A. Olivares Pino, D. Oliveira Damazio, E. Oliver Garcia, D. Olivito, A. Olszewski, J. Olszowska, A. Onofre, P. U. E. Onyisi, C. J. Oram, M. J. Oreglia, Y. Oren, D. Orestano, N. Orlando, C. Oropeza Barrera, R. S. Orr, B. Osculati, R. Ospanov, G. Otero y Garzon, H. Otono, M. Ouchrif, E. A. Ouellette, F. Ould-Saada, A. Ouraou, K. P. Oussoren, Q. Ouyang, A. Ovcharova, M. Owen, V. E. Ozcan, N. Ozturk, K. Pachal, A. Pacheco Pages, C. Padilla Aranda, S. Pagan Griso, E. Paganis, C. Pahl, F. Paige, P. Pais, K. Pajchel, G. Palacino, S. Palestini, D. Pallin, A. Palma, J. D. Palmer, Y. B. Pan, E. Panagiotopoulou, J. G. Panduro Vazquez, P. Pani, N. Panikashvili, S. Panitkin, D. Pantea, Th. D. Papadopoulou, K. Papageorgiou, A. Paramonov, D. Paredes Hernandez, M. A. Parker, F. Parodi, J. A. Parsons, U. Parzefall, E. Pasqualucci, S. Passaggio, A. Passeri, F. Pastore, Fr. Pastore, G. Pásztor, S. Pataraia, N. D. Patel, J. R. Pater, S. Patricelli, T. Pauly, J. Pearce, M. Pedersen, S. Pedraza Lopez, R. Pedro, S. V. Peleganchuk, D. Pelikan, H. Peng, B. Penning, J. Penwell, D. V. Perepelitsa, E. Perez Codina, M. T. Pérez García-Estañ, V. Perez Reale, L. Perini, H. Pernegger, R. Perrino, R. Peschke, V. D. Peshekhonov, K. Peters, R. F. Y. Peters, B. A. Petersen, J. Petersen, T. C. Petersen, E. Petit, A. Petridis, C. Petridou, E. Petrolo, F. Petrucci, M. Petteni, R. Pezoa, P. W. Phillips, G. Piacquadio, E. Pianori, A. Picazio, E. Piccaro, M. Piccinini, S. M. Piec, R. Piegaia, D. T. Pignotti, J. E. Pilcher, A. D. Pilkington, J. Pina, M. Pinamonti, A. Pinder, J. L. Pinfold, A. Pingel, B. Pinto, C. Pizio, M.-A. Pleier, V. Pleskot, E. Plotnikova, P. Plucinski, S. Poddar, F. Podlyski, R. Poettgen, L. Poggioli, D. Pohl, M. Pohl, G. Polesello, A. Policicchio, R. Polifka, A. Polini, C. S. Pollard, V. Polychronakos, K. Pommès, L. Pontecorvo, B. G. Pope, G. A. Popeneciu, D. S. Popovic, A. Poppleton, X. Portell Bueso, G. E. Pospelov, S. Pospisil, K. Potamianos, I. N. Potrap, C. J. Potter, C. T. Potter, G. Poulard, J. Poveda, V. Pozdnyakov, R. Prabhu, P. Pralavorio, A. Pranko, S. Prasad, R. Pravahan, S. Prell, D. Price, J. Price, L. E. Price, D. Prieur, M. Primavera, M. Proissl, K. Prokofiev, F. Prokoshin, E. Protopapadaki, S. Protopopescu, J. Proudfoot, M. Przybycien, H. Przysiezniak, E. Ptacek, E. Pueschel, D. Puldon, M. Purohit, P. Puzo, Y. Pylypchenko, J. Qian, A. Quadt, D. R. Quarrie, W. B. Quayle, D. Quilty, A. Qureshi, V. Radeka, V. Radescu, S. K. Radhakrishnan, P. Radloff, F. Ragusa, G. Rahal, S. Rajagopalan, M. Rammensee, M. Rammes, A. S. Randle-Conde, C. Rangel-Smith, K. Rao, F. Rauscher, T. C. Rave, T. Ravenscroft, M. Raymond, A. L. Read, D. M. Rebuzzi, A. Redelbach, G. Redlinger, R. Reece, K. Reeves, L. Rehnisch, A. Reinsch, H. Reisin, M. Relich, C. Rembser, Z. L. Ren, A. Renaud, M. Rescigno, S. Resconi, O. L. Rezanova, P. Reznicek, R. Rezvani, R. Richter, M. Ridel, P. Rieck, M. Rijssenbeek, A. Rimoldi, L. Rinaldi, E. Ritsch, I. Riu, F. Rizatdinova, E. Rizvi, S. H. Robertson, A. Robichaud-Veronneau, D. Robinson, J. E. M. Robinson, A. Robson, C. Roda, D. Roda Dos Santos, L. Rodrigues, S. Roe, O. Røhne, S. Rolli, A. Romaniouk, M. Romano, G. Romeo, E. Romero Adam, N. Rompotis, L. Roos, E. Ros, S. Rosati, K. Rosbach, A. Rose, M. Rose, P. L. Rosendahl, O. Rosenthal, V. Rossetti, E. Rossi, L. P. Rossi, R. Rosten, M. Rotaru, I. Roth, J. Rothberg, D. Rousseau, C. R. Royon, A. Rozanov, Y. Rozen, X. Ruan, F. Rubbo, I. Rubinskiy, V. I. Rud, C. Rudolph, M. S. Rudolph, F. Rühr, A. Ruiz-Martinez, Z. Rurikova, N. A. Rusakovich, A. Ruschke, J. P. Rutherfoord, N. Ruthmann, P. Ruzicka, Y. F. Ryabov, M. Rybar, G. Rybkin, N. C. Ryder, A. F. Saavedra, S. Sacerdoti, A. Saddique, I. Sadeh, H. F-W. Sadrozinski, R. Sadykov, F. Safai Tehrani, H. Sakamoto, Y. Sakurai, G. Salamanna, A. Salamon, M. Saleem, D. Salek, P. H. Sales De Bruin, D. Salihagic, A. Salnikov, J. Salt, B. M. Salvachua Ferrando, D. Salvatore, F. Salvatore, A. Salvucci, A. Salzburger, D. Sampsonidis, A. Sanchez, J. Sánchez, V. Sanchez Martinez, H. Sandaker, H. G. Sander, M. P. Sanders, M. Sandhoff, T. Sandoval, C. Sandoval, R. Sandstroem, D. P. C. Sankey, A. Sansoni, C. Santoni, R. Santonico, H. Santos, I. Santoyo Castillo, K. Sapp, A. Sapronov, J. G. Saraiva, B. Sarrazin, G. Sartisohn, O. Sasaki, Y. Sasaki, G. Sauvage, E. Sauvan, J. B. Sauvan, P. Savard, D. O. Savu, C. Sawyer, L. Sawyer, D. H. Saxon, J. Saxon, C. Sbarra, A. Sbrizzi, T. Scanlon, D. A. Scannicchio, M. Scarcella, J. Schaarschmidt, P. Schacht, D. Schaefer, A. Schaelicke, S. Schaepe, S. Schaetzel, U. Schäfer, A. C. Schaffer, D. Schaile, R. D. Schamberger, V. Scharf, V. A. Schegelsky, D. Scheirich, M. Schernau, M. I. Scherzer, C. Schiavi, J. Schieck, C. Schillo, M. Schioppa, S. Schlenker, E. Schmidt, K. Schmieden, C. Schmitt, S. Schmitt, B. Schneider, Y. J. Schnellbach, U. Schnoor, L. Schoeffel, A. Schoening, B. D. Schoenrock, A. L. S. Schorlemmer, M. Schott, D. Schouten, J. Schovancova, S. Schramm, M. Schreyer, C. Schroeder, N. Schuh, M. J. Schultens, H.-C. Schultz-Coulon, H. Schulz, M. Schumacher, B. A. Schumm, Ph. Schune, A. Schwartzman, Ph. Schwegler, Ph. Schwemling, R. Schwienhorst, J. Schwindling, T. Schwindt, M. Schwoerer, F. G. Sciacca, E. Scifo, G. Sciolla, W. G. Scott, F. Scuri, F. Scutti, J. Searcy, G. Sedov, E. Sedykh, S. C. Seidel, A. Seiden, F. Seifert, J. M. Seixas, G. Sekhniaidze, S. J. Sekula, K. E. Selbach, D. M. Seliverstov, G. Sellers, M. Seman, N. Semprini-Cesari, C. Serfon, L. Serin, L. Serkin, T. Serre, R. Seuster, H. Severini, F. Sforza, A. Sfyrla, E. Shabalina, M. Shamim, L. Y. Shan, J. T. Shank, Q. T. Shao, M. Shapiro, P. B. Shatalov, K. Shaw, P. Sherwood, S. Shimizu, C. O. Shimmin, M. Shimojima, M. Shiyakova, A. Shmeleva, M. J. Shochet, D. Short, S. Shrestha, E. Shulga, M. A. Shupe, S. Shushkevich, P. Sicho, D. Sidorov, A. Sidoti, F. Siegert, Dj. Sijacki, O. Silbert, J. Silva, Y. Silver, D. Silverstein, S. B. Silverstein, V. Simak, O. Simard, Lj. Simic, S. Simion, E. Simioni, B. Simmons, R. Simoniello, M. Simonyan, P. Sinervo, N. B. Sinev, V. Sipica, G. Siragusa, A. Sircar, A. N. Sisakyan, S. Yu. Sivoklokov, J. Sjölin, T. B. Sjursen, L. A. Skinnari, H. P. Skottowe, K. Yu. Skovpen, P. Skubic, M. Slater, T. Slavicek, K. Sliwa, V. Smakhtin, B. H. Smart, L. Smestad, S. Yu. Smirnov, Y. Smirnov, L. N. Smirnova, O. Smirnova, K. M. Smith, M. Smizanska, K. Smolek, A. A. Snesarev, G. Snidero, S. Snyder, R. Sobie, F. Socher, A. Soffer, D. A. Soh, C. A. Solans, M. Solar, J. Solc, E. Yu. Soldatov, U. Soldevila, E. Solfaroli Camillocci, A. A. Solodkov, O. V. Solovyanov, V. Solovyev, P. Sommer, N. Soni, A. Sood, B. Sopko, V. Sopko, M. Sosebee, R. Soualah, P. Soueid, A. M. Soukharev, D. South, S. Spagnolo, F. Spanò, W. R. Spearman, R. Spighi, G. Spigo, M. Spousta, T. Spreitzer, B. Spurlock, R. D. St. Denis, J. Stahlman, R. Stamen, E. Stanecka, R. W. Stanek, C. Stanescu, M. Stanescu-Bellu, M. M. Stanitzki, S. Stapnes, E. A. Starchenko, J. Stark, P. Staroba, P. Starovoitov, R. Staszewski, P. Stavina, G. Steele, P. Steinberg, B. Stelzer, H. J. Stelzer, O. Stelzer-Chilton, H. Stenzel, S. Stern, G. A. Stewart, J. A. Stillings, M. C. Stockton, M. Stoebe, K. Stoerig, G. Stoicea, S. Stonjek, A. R. Stradling, A. Straessner, J. Strandberg, S. Strandberg, A. Strandlie, E. Strauss, M. Strauss, P. Strizenec, R. Ströhmer, D. M. Strom, R. Stroynowski, S. A. Stucci, B. Stugu, I. Stumer, N. A. Styles, D. Su, J. Su, HS. Subramania, R. Subramaniam, A. Succurro, Y. Sugaya, C. Suhr, M. Suk, V. V. Sulin, S. Sultansoy, T. Sumida, X. Sun, J. E. Sundermann, K. Suruliz, G. Susinno, M. R. Sutton, Y. Suzuki, M. Svatos, S. Swedish, M. Swiatlowski, I. Sykora, T. Sykora, D. Ta, K. Tackmann, J. Taenzer, A. Taffard, R. Tafirout, N. Taiblum, Y. Takahashi, H. Takai, R. Takashima, H. Takeda, T. Takeshita, Y. Takubo, M. Talby, A. A. Talyshev, J. Y. C. Tam, M. C. Tamsett, K. G. Tan, J. Tanaka, R. Tanaka, S. Tanaka, S. Tanaka, A. J. Tanasijczuk, K. Tani, N. Tannoury, S. Tapprogge, S. Tarem, F. Tarrade, G. F. Tartarelli, P. Tas, M. Tasevsky, T. Tashiro, E. Tassi, A. Tavares Delgado, Y. Tayalati, C. Taylor, F. E. Taylor, G. N. Taylor, W. Taylor, F. A. Teischinger, M. Teixeira Dias Castanheira, P. Teixeira-Dias, K. K. Temming, H. Ten Kate, P. K. Teng, S. Terada, K. Terashi, J. Terron, S. Terzo, M. Testa, R. J. Teuscher, J. Therhaag, T. Theveneaux-Pelzer, S. Thoma, J. P. Thomas, J. Thomas-Wilsker, E. N. Thompson, P. D. Thompson, P. D. Thompson, R. J. Thompson, A. S. Thompson, L. A. Thomsen, E. Thomson, M. Thomson, W. M. Thong, R. P. Thun, F. Tian, M. J. Tibbetts, V. O. Tikhomirov, Yu. A. Tikhonov, S. Timoshenko, E. Tiouchichine, P. Tipton, S. Tisserant, T. Todorov, S. Todorova-Nova, B. Toggerson, J. Tojo, S. Tokár, K. Tokushuku, K. Tollefson, L. Tomlinson, M. Tomoto, L. Tompkins, K. Toms, N. D. Topilin, E. Torrence, H. Torres, E. Torró Pastor, J. Toth, F. Touchard, D. R. Tovey, H. L. Tran, T. Trefzger, L. Tremblet, A. Tricoli, I. M. Trigger, S. Trincaz-Duvoid, M. F. Tripiana, N. Triplett, W. Trischuk, B. Trocmé, C. Troncon, M. Trottier-McDonald, M. Trovatelli, P. True, M. Trzebinski, A. Trzupek, C. Tsarouchas, J. C-L. Tseng, P. V. Tsiareshka, D. Tsionou, G. Tsipolitis, N. Tsirintanis, S. Tsiskaridze, V. Tsiskaridze, E. G. Tskhadadze, I. I. Tsukerman, V. Tsulaia, S. Tsuno, D. Tsybychev, A. Tua, A. Tudorache, V. Tudorache, A. N. Tuna, S. A. Tupputi, S. Turchikhin, D. Turecek, R. Turra, P. M. Tuts, A. Tykhonov, M. Tylmad, M. Tyndel, K. Uchida, I. Ueda, R. Ueno, M. Ughetto, M. Ugland, M. Uhlenbrock, F. Ukegawa, G. Unal, A. Undrus, G. Unel, F. C. Ungaro, Y. Unno, C. Unverdorben, D. Urbaniec, P. Urquijo, G. Usai, A. Usanova, L. Vacavant, V. Vacek, B. Vachon, N. Valencic, S. Valentinetti, A. Valero, L. Valery, S. Valkar, E. Valladolid Gallego, S. Vallecorsa, J. A. Valls Ferrer, P. C. Van Der Deijl, R. van der Geer, H. van der Graaf, R. Van Der Leeuw, D. van der Ster, N. van Eldik, P. van Gemmeren, J. Van Nieuwkoop, I. van Vulpen, M. C. van Woerden, M. Vanadia, W. Vandelli, A. Vaniachine, F. Vannucci, G. Vardanyan, R. Vari, E. W. Varnes, T. Varol, D. Varouchas, A. Vartapetian, K. E. Varvell, F. Vazeille, T. Vazquez Schroeder, J. Veatch, F. Veloso, T. Velz, S. Veneziano, A. Ventura, D. Ventura, M. Venturi, N. Venturi, A. Venturini, V. Vercesi, M. Verducci, W. Verkerke, J. C. Vermeulen, A. Vest, M. C. Vetterli, O. Viazlo, I. Vichou, T. Vickey, O. E. Vickey Boeriu, G. H. A. Viehhauser, S. Viel, R. Vigne, M. Villa, M. Villaplana Perez, E. Vilucchi, M. G. Vincter, V. B. Vinogradov, J. Virzi, O. Vitells, I. Vivarelli, F. Vives Vaque, S. Vlachos, D. Vladoiu, M. Vlasak, A. Vogel, P. Vokac, G. Volpi, M. Volpi, H. von der Schmitt, H. von Radziewski, E. von Toerne, V. Vorobel, M. Vos, R. Voss, J. H. Vossebeld, N. Vranjes, M. Vranjes Milosavljevic, V. Vrba, M. Vreeswijk, T. Vu Anh, R. Vuillermet, I. Vukotic, Z. Vykydal, P. Wagner, W. Wagner, S. Wahrmund, J. Wakabayashi, J. Walder, R. Walker, W. Walkowiak, R. Wall, P. Waller, B. Walsh, C. Wang, C. Wang, F. Wang, H. Wang, H. Wang, J. Wang, J. Wang, K. Wang, R. Wang, S. M. Wang, T. Wang, X. Wang, A. Warburton, C. P. Ward, D. R. Wardrope, M. Warsinsky, A. Washbrook, C. Wasicki, I. Watanabe, P. M. Watkins, A. T. Watson, I. J. Watson, M. F. Watson, G. Watts, S. Watts, A. T. Waugh, B. M. Waugh, S. Webb, M. S. Weber, S. W. Weber, J. S. Webster, A. R. Weidberg, P. Weigell, J. Weingarten, C. Weiser, H. Weits, P. S. Wells, T. Wenaus, D. Wendland, Z. Weng, T. Wengler, S. Wenig, N. Wermes, M. Werner, P. Werner, M. Wessels, J. Wetter, K. Whalen, A. White, M. J. White, R. White, S. White, D. Whiteson, D. Wicke, F. J. Wickens, W. Wiedenmann, M. Wielers, P. Wienemann, C. Wiglesworth, L. A. M. Wiik-Fuchs, P. A. Wijeratne, A. Wildauer, M. A. Wildt, H. G. Wilkens, J. Z. Will, H. H. Williams, S. Williams, S. Willocq, A. Wilson, J. A. Wilson, I. Wingerter-Seez, S. Winkelmann, F. Winklmeier, M. Wittgen, T. Wittig, J. Wittkowski, S. J. Wollstadt, M. W. Wolter, H. Wolters, B. K. Wosiek, J. Wotschack, M. J. Woudstra, K. W. Wozniak, M. Wright, S. L. Wu, X. Wu, Y. Wu, E. Wulf, T. R. Wyatt, B. M. Wynne, S. Xella, M. Xiao, D. Xu, L. Xu, B. Yabsley, S. Yacoob, M. Yamada, H. Yamaguchi, Y. Yamaguchi, A. Yamamoto, K. Yamamoto, S. Yamamoto, T. Yamamura, T. Yamanaka, K. Yamauchi, Y. Yamazaki, Z. Yan, H. Yang, H. Yang, U. K. Yang, Y. Yang, S. Yanush, L. Yao, Y. Yasu, E. Yatsenko, K. H. Yau Wong, J. Ye, S. Ye, A. L. Yen, E. Yildirim, M. Yilmaz, R. Yoosoofmiya, K. Yorita, R. Yoshida, K. Yoshihara, C. Young, C. J. S. Young, S. Youssef, D. R. Yu, J. Yu, J. M. Yu, J. Yu, L. Yuan, A. Yurkewicz, B. Zabinski, R. Zaidan, A. M. Zaitsev, A. Zaman, S. Zambito, L. Zanello, D. Zanzi, A. Zaytsev, C. Zeitnitz, M. Zeman, A. Zemla, K. Zengel, O. Zenin, T. Ženiš, D. Zerwas, G. Zevi della Porta, D. Zhang, F. Zhang, H. Zhang, J. Zhang, L. Zhang, X. Zhang, Z. Zhang, Z. Zhao, A. Zhemchugov, J. Zhong, B. Zhou, L. Zhou, N. Zhou, C. G. Zhu, H. Zhu, J. Zhu, Y. Zhu, X. Zhuang, A. Zibell, D. Zieminska, N. I. Zimine, C. Zimmermann, R. Zimmermann, S. Zimmermann, S. Zimmermann, Z. Zinonos, M. Ziolkowski, R. Zitoun, G. Zobernig, A. Zoccoli, M. zur Nedden, G. Zurzolo, V. Zutshi, L. Zwalinski, ATLAS Collaboration

**Affiliations:** 1 Department of Physics, University of Adelaide, Adelaide, Australia; 2 Physics Department, SUNY Albany, Albany, NY USA; 3 Department of Physics, University of Alberta, Edmonton, AB Canada; 4 Department of Physics, Ankara University, Ankara, Turkey; 5 Department of Physics, Gazi University, Istanbul, Turkey; 6 Division of Physics, TOBB University of Economics and Technology, Ankara, Turkey; 7 Turkish Atomic Energy Authority, Ankara, Turkey; 8 LAPP, CNRS/IN2P3 and Université Savoie Mont Blanc, Annecy-le-Vieux, France; 9 High Energy Physics Division, Argonne National Laboratory, Argonne, IL USA; 10 Department of Physics, University of Arizona, Tucson, AZ USA; 11 Department of Physics, The University of Texas at Arlington, Arlington, TX USA; 12 Physics Department, University of Athens, Athens, Greece; 13 Physics Department, National Technical University of Athens, Zografou, Greece; 14 Institute of Physics, Azerbaijan Academy of Sciences, Baku, Azerbaijan; 15 Institut de Física d’Altes Energies and Departament de Física de la Universitat Autònoma de Barcelona, Barcelona, Spain; 16 Institute of Physics, University of Belgrade, Belgrade, Serbia; 17 Department for Physics and Technology, University of Bergen, Bergen, Norway; 18 Physics Division, Lawrence Berkeley National Laboratory and University of California, Berkeley, CA USA; 19 Department of Physics, Humboldt University, Berlin, Germany; 20 Albert Einstein Center for Fundamental Physics and Laboratory for High Energy Physics, University of Bern, Bern, Switzerland; 21 School of Physics and Astronomy, University of Birmingham, Birmingham, UK; 22 Department of Physics, Bogazici University, Istanbul, Turkey; 23 Department of Physics Engineering, Dogus University, Istanbul, Turkey; 24 Department of Physics Engineering, Gaziantep University, Gaziantep, Turkey; 25 INFN Sezione di Bologna, Bologna, Italy; 26 Dipartimento di Fisica e Astronomia, Università di Bologna, Bologna, Italy; 27 Physikalisches Institut, University of Bonn, Bonn, Germany; 28 Department of Physics, Boston University, Boston, MA USA; 29 Department of Physics, Brandeis University, Waltham, MA USA; 30 Universidade Federal do Rio De Janeiro COPPE/EE/IF, Rio de Janeiro, Brazil; 31 Electrical Circuits Department, Federal University of Juiz de Fora (UFJF), Juiz de Fora, Brazil; 32 Federal University of Sao Joao del Rei (UFSJ), Sao Joao del Rei, Brazil; 33 Instituto de Fisica, Universidade de Sao Paulo, Sao Paulo, Brazil; 34 Physics Department, Brookhaven National Laboratory, Upton, NY USA; 35 National Institute of Physics and Nuclear Engineering, Bucharest, Romania; 36 Physics Department, National Institute for Research and Development of Isotopic and Molecular Technologies, Cluj Napoca, Romania; 37 University Politehnica Bucharest, Bucharest, Romania; 38 West University in Timisoara, Timisoara, Romania; 39 Departamento de Física, Universidad de Buenos Aires, Buenos Aires, Argentina; 40 Cavendish Laboratory, University of Cambridge, Cambridge, UK; 41 Department of Physics, Carleton University, Ottawa, ON Canada; 42 CERN, Geneva, Switzerland; 43 Enrico Fermi Institute, University of Chicago, Chicago, IL USA; 44 Departamento de Física, Pontificia Universidad Católica de Chile, Santiago, Chile; 45 Departamento de Física, Universidad Técnica Federico Santa María, Valparaiso, Chile; 46 Institute of High Energy Physics, Chinese Academy of Sciences, Beijing, China; 47 Department of Modern Physics, University of Science and Technology of China, Hefei, Anhui China; 48 Department of Physics, Nanjing University, Jiangsu, China; 49 School of Physics, Shandong University, Shandong, China; 50 Shanghai Key Laboratory for Particle Physics and Cosmology, Department of Physics and Astronomy, Shanghai Jiao Tong University, Shanghai, China; 51 Laboratoire de Physique Corpusculaire, Clermont Université and Université Blaise Pascal and CNRS/IN2P3, Clermont-Ferrand, France; 52 Nevis Laboratory, Columbia University, Irvington, NY USA; 53 Niels Bohr Institute, University of Copenhagen, Copenhagen, Denmark; 54 INFN Gruppo Collegato di Cosenza, Laboratori Nazionali di Frascati, Frascati, Italy; 55 Dipartimento di Fisica, Università della Calabria, Rende, Italy; 56 AGH University of Science and Technology, Faculty of Physics and Applied Computer Science, Krakow, Poland; 57 Marian Smoluchowski Institute of Physics, Jagiellonian University, Krakow, Poland; 58 Institute of Nuclear Physics, Polish Academy of Sciences, Krakow, Poland; 59 Physics Department, Southern Methodist University, Dallas, TX USA; 60 Physics Department, University of Texas at Dallas, Richardson, TX USA; 61 DESY, Hamburg and Zeuthen, Germany; 62 Institut für Experimentelle Physik IV, Technische Universität Dortmund, Dortmund, Germany; 63 Institut für Kern- und Teilchenphysik, Technische Universität Dresden, Dresden, Germany; 64 Department of Physics, Duke University, Durham, NC USA; 65 SUPA-School of Physics and Astronomy, University of Edinburgh, Edinburgh, UK; 66 INFN Laboratori Nazionali di Frascati, Frascati, Italy; 67 Fakultät für Mathematik und Physik, Albert-Ludwigs-Universität, Freiburg, Germany; 68 Section de Physique, Université de Genève, Geneva, Switzerland; 69 INFN Sezione di Genova, Genoa, Italy; 70 Dipartimento di Fisica, Università di Genova, Genoa, Italy; 71 E. Andronikashvili Institute of Physics, Iv. Javakhishvili Tbilisi State University, Tbilisi, Georgia; 72 High Energy Physics Institute, Tbilisi State University, Tbilisi, Georgia; 73 II Physikalisches Institut, Justus-Liebig-Universität Giessen, Giessen, Germany; 74 SUPA-School of Physics and Astronomy, University of Glasgow, Glasgow, UK; 75 II Physikalisches Institut, Georg-August-Universität, Göttingen, Germany; 76 Laboratoire de Physique Subatomique et de Cosmologie, Université Grenoble-Alpes, CNRS/IN2P3, Grenoble, France; 77 Department of Physics, Hampton University, Hampton, VA USA; 78 Laboratory for Particle Physics and Cosmology, Harvard University, Cambridge, MA USA; 79 Kirchhoff-Institut für Physik, Ruprecht-Karls-Universität Heidelberg, Heidelberg, Germany; 80 Physikalisches Institut, Ruprecht-Karls-Universität Heidelberg, Heidelberg, Germany; 81 ZITI Institut für technische Informatik, Ruprecht-Karls-Universität Heidelberg, Mannheim, Germany; 82 Faculty of Applied Information Science, Hiroshima Institute of Technology, Hiroshima, Japan; 83 Department of Physics, Indiana University, Bloomington, IN USA; 84 Institut für Astro- und Teilchenphysik, Leopold-Franzens-Universität, Innsbruck, Austria; 85 University of Iowa, Iowa City, IA USA; 86 Department of Physics and Astronomy, Iowa State University, Ames, IA USA; 87 Joint Institute for Nuclear Research, JINR Dubna, Dubna, Russia; 88 KEK, High Energy Accelerator Research Organization, Tsukuba, Japan; 89 Graduate School of Science, Kobe University, Kobe, Japan; 90 Faculty of Science, Kyoto University, Kyoto, Japan; 91 Kyoto University of Education, Kyoto, Japan; 92 Department of Physics, Kyushu University, Fukuoka, Japan; 93 Instituto de Física La Plata, Universidad Nacional de La Plata and CONICET, La Plata, Argentina; 94 Physics Department, Lancaster University, Lancaster, UK; 95 INFN Sezione di Lecce, Lecce, Italy; 96 Dipartimento di Matematica e Fisica, Università del Salento, Lecce, Italy; 97 Oliver Lodge Laboratory, University of Liverpool, Liverpool, UK; 98 Department of Physics, Jožef Stefan Institute and University of Ljubljana, Ljubljana, Slovenia; 99 School of Physics and Astronomy, Queen Mary University of London, London, UK; 100 Department of Physics, Royal Holloway University of London, Surrey, UK; 101 Department of Physics and Astronomy, University College London, London, UK; 102 Louisiana Tech University, Ruston, LA USA; 103 Laboratoire de Physique Nucléaire et de Hautes Energies, UPMC and Université Paris-Diderot and CNRS/IN2P3, Paris, France; 104 Fysiska institutionen, Lunds universitet, Lund, Sweden; 105 Departamento de Fisica Teorica C-15, Universidad Autonoma de Madrid, Madrid, Spain; 106 Institut für Physik, Universität Mainz, Mainz, Germany; 107 School of Physics and Astronomy, University of Manchester, Manchester, UK; 108 CPPM, Aix-Marseille Université and CNRS/IN2P3, Marseille, France; 109 Department of Physics, University of Massachusetts, Amherst, MA USA; 110 Department of Physics, McGill University, Montreal, QC Canada; 111 School of Physics, University of Melbourne, Melbourne, VIC Australia; 112 Department of Physics, The University of Michigan, Ann Arbor, MI USA; 113 Department of Physics and Astronomy, Michigan State University, East Lansing, MI USA; 114 INFN Sezione di Milano, Milan, Italy; 115 Dipartimento di Fisica, Università di Milano, Milan, Italy; 116 B.I. Stepanov Institute of Physics, National Academy of Sciences of Belarus, Minsk, Republic of Belarus; 117 National Scientific and Educational Centre for Particle and High Energy Physics, Minsk, Republic of Belarus; 118 Department of Physics, Massachusetts Institute of Technology, Cambridge, MA USA; 119 Group of Particle Physics, University of Montreal, Montreal, QC Canada; 120 P.N. Lebedev Institute of Physics, Academy of Sciences, Moscow, Russia; 121 Institute for Theoretical and Experimental Physics (ITEP), Moscow, Russia; 122 National Research Nuclear University MEPhI, Moscow, Russia; 123 D.V. Skobeltsyn Institute of Nuclear Physics, M.V. Lomonosov Moscow State University, Moscow, Russia; 124 Fakultät für Physik, Ludwig-Maximilians-Universität München, Munich, Germany; 125 Max-Planck-Institut für Physik (Werner-Heisenberg-Institut), München, Germany; 126 Nagasaki Institute of Applied Science, Nagasaki, Japan; 127 Graduate School of Science and Kobayashi-Maskawa Institute, Nagoya University, Nagoya, Japan; 128 INFN Sezione di Napoli, Naples, Italy; 129 Dipartimento di Fisica, Università di Napoli, Naples, Italy; 130 Department of Physics and Astronomy, University of New Mexico, Albuquerque, NM USA; 131 Institute for Mathematics, Astrophysics and Particle Physics, Radboud University Nijmegen/Nikhef, Nijmegen, The Netherlands; 132 Nikhef National Institute for Subatomic Physics and University of Amsterdam, Amsterdam, The Netherlands; 133 Department of Physics, Northern Illinois University, De Kalb, IL USA; 134 Budker Institute of Nuclear Physics, SB RAS, Novosibirsk, Russia; 135 Department of Physics, New York University, New York, NY USA; 136 Ohio State University, Columbus, OH USA; 137 Faculty of Science, Okayama University, Okayama, Japan; 138 Homer L. Dodge Department of Physics and Astronomy, University of Oklahoma, Norman, OK USA; 139 Department of Physics, Oklahoma State University, Stillwater, OK USA; 140 Palacký University, RCPTM, Olomouc, Czech Republic; 141 Center for High Energy Physics, University of Oregon, Eugene, OR USA; 142 LAL, Université Paris-Sud and CNRS/IN2P3, Orsay, France; 143 Graduate School of Science, Osaka University, Osaka, Japan; 144 Department of Physics, University of Oslo, Oslo, Norway; 145 Department of Physics, Oxford University, Oxford, UK; 146 INFN Sezione di Pavia, Pavia, Italy; 147 Dipartimento di Fisica, Università di Pavia, Pavia, Italy; 148 Department of Physics, University of Pennsylvania, Philadelphia, PA USA; 149 National Research Centre “Kurchatov Institute” B.P.Konstantinov Petersburg Nuclear Physics Institute, St. Petersburg, Russia; 150 INFN Sezione di Pisa, Pisa, Italy; 151 Dipartimento di Fisica E. Fermi, Università di Pisa, Pisa, Italy; 152 Department of Physics and Astronomy, University of Pittsburgh, Pittsburgh, PA USA; 153 Laboratório de Instrumentação e Física Experimental de Partículas-LIP, Lisbon, Portugal; 154 Faculdade de Ciências, Universidade de Lisboa, Lisbon, Portugal; 155 Department of Physics, University of Coimbra, Coimbra, Portugal; 156 Centro de Física Nuclear da Universidade de Lisboa, Lisbon, Portugal; 157 Departamento de Fisica, Universidade do Minho, Braga, Portugal; 158 Departamento de Fisica Teorica y del Cosmos and CAFPE, Universidad de Granada, Granada, Spain; 159 Dep Fisica and CEFITEC of Faculdade de Ciencias e Tecnologia, Universidade Nova de Lisboa, Caparica, Portugal; 160 Institute of Physics, Academy of Sciences of the Czech Republic, Prague, Czech Republic; 161 Czech Technical University in Prague, Prague, Czech Republic; 162 Faculty of Mathematics and Physics, Charles University in Prague, Prague, Czech Republic; 163 State Research Center Institute for High Energy Physics, Protvino, Russia; 164 Particle Physics Department, Rutherford Appleton Laboratory, Didcot, UK; 165 Physics Department, University of Regina, Regina SK, Canada; 166 Ritsumeikan University, Kusatsu, Shiga Japan; 167 INFN Sezione di Roma, Rome, Italy; 168 Dipartimento di Fisica, Sapienza Università di Roma, Rome, Italy; 169 INFN Sezione di Roma Tor Vergata, Rome, Italy; 170 Dipartimento di Fisica, Università di Roma Tor Vergata, Rome, Italy; 171 INFN Sezione di Roma Tre, Rome, Italy; 172 Dipartimento di Matematica e Fisica, Università Roma Tre, Rome, Italy; 173 Faculté des Sciences Ain Chock, Réseau Universitaire de Physique des Hautes Energies-Université Hassan II, Casablanca, Morocco; 174 Centre National de l’Energie des Sciences Techniques Nucleaires, Rabat, Morocco; 175 Faculté des Sciences Semlalia, Université Cadi Ayyad, LPHEA-Marrakech, Marrakech, Morocco; 176 Faculté des Sciences, Université Mohamed Premier and LPTPM, Oujda, Morocco; 177 Faculté des Sciences, Université Mohammed V-Agdal, Rabat, Morocco; 178 DSM/IRFU (Institut de Recherches sur les Lois Fondamentales de l’Univers), CEA Saclay (Commissariat à l’Energie Atomique et aux Energies Alternatives), Gif-sur-Yvette, France; 179 Santa Cruz Institute for Particle Physics, University of California Santa Cruz, Santa Cruz, CA USA; 180 Department of Physics, University of Washington, Seattle, WA USA; 181 Department of Physics and Astronomy, University of Sheffield, Sheffield, UK; 182 Department of Physics, Shinshu University, Nagano, Japan; 183 Fachbereich Physik, Universität Siegen, Siegen, Germany; 184 Department of Physics, Simon Fraser University, Burnaby, BC Canada; 185 SLAC National Accelerator Laboratory, Stanford, CA USA; 186 Faculty of Mathematics, Physics and Informatics, Comenius University, Bratislava, Slovak Republic; 187 Department of Subnuclear Physics, Institute of Experimental Physics of the Slovak Academy of Sciences, Kosice, Slovak Republic; 188 Department of Physics, University of Cape Town, Cape Town, South Africa; 189 Department of Physics, University of Johannesburg, Johannesburg, South Africa; 190 School of Physics, University of the Witwatersrand, Johannesburg, South Africa; 191 Department of Physics, Stockholm University, Stockholm, Sweden; 192 The Oskar Klein Centre, Stockholm, Sweden; 193 Physics Department, Royal Institute of Technology, Stockholm, Sweden; 194 Departments of Physics and Astronomy and Chemistry, Stony Brook University, Stony Brook, NY USA; 195 Department of Physics and Astronomy, University of Sussex, Brighton, UK; 196 School of Physics, University of Sydney, Sydney, Australia; 197 Institute of Physics, Academia Sinica, Taipei, Taiwan; 198 Department of Physics, Technion: Israel Institute of Technology, Haifa, Israel; 199 Raymond and Beverly Sackler School of Physics and Astronomy, Tel Aviv University, Tel Aviv, Israel; 200 Department of Physics, Aristotle University of Thessaloniki, Thessaloníki, Greece; 201 International Center for Elementary Particle Physics and Department of Physics, The University of Tokyo, Tokyo, Japan; 202 Graduate School of Science and Technology, Tokyo Metropolitan University, Tokyo, Japan; 203 Department of Physics, Tokyo Institute of Technology, Tokyo, Japan; 204 Department of Physics, University of Toronto, Toronto, ON Canada; 205 TRIUMF, Vancouver, BC Canada; 206 Department of Physics and Astronomy, York University, Toronto, ON Canada; 207 Faculty of Pure and Applied Sciences, University of Tsukuba, Tsukuba, Japan; 208 Department of Physics and Astronomy, Tufts University, Medford, MA USA; 209 Centro de Investigaciones, Universidad Antonio Narino, Bogotá, Colombia; 210 Department of Physics and Astronomy, University of California Irvine, Irvine, CA USA; 211 INFN Gruppo Collegato di Udine, Sezione di Trieste, Udine, Italy; 212 ICTP, Trieste, Italy; 213 Dipartimento di Chimica Fisica e Ambiente, Università di Udine, Udine, Italy; 214 Department of Physics, University of Illinois, Urbana, IL USA; 215 Department of Physics and Astronomy, University of Uppsala, Uppsala, Sweden; 216 Instituto de Física Corpuscular (IFIC) and Departamento de Física Atómica, Molecular y Nuclear and Departamento de Ingeniería Electrónica and Instituto de Microelectrónica de Barcelona (IMB-CNM), University of Valencia and CSIC, Valencia, Spain; 217 Department of Physics, University of British Columbia, Vancouver, BC Canada; 218 Department of Physics and Astronomy, University of Victoria, Victoria, BC Canada; 219 Department of Physics, University of Warwick, Coventry, UK; 220 Waseda University, Tokyo, Japan; 221 Department of Particle Physics, The Weizmann Institute of Science, Rehovot, Israel; 222 Department of Physics, University of Wisconsin, Madison, WI USA; 223 Fakultät für Physik und Astronomie, Julius-Maximilians-Universität, Würzburg, Germany; 224 Fachbereich C Physik, Bergische Universität Wuppertal, Wuppertal, Germany; 225 Department of Physics, Yale University, New Haven, CT USA; 226 Yerevan Physics Institute, Yerevan, Armenia; 227 Centre de Calcul de l’Institut National de Physique Nucléaire et de Physique des Particules (IN2P3), Villeurbanne, France; 228 CERN, 1211 Geneva 23, Switzerland

## Abstract

The centrality dependence of the mean charged-particle multiplicity as a function of pseudorapidity is measured in approximately 1 $\upmu$b$^{-1}$ of proton–lead collisions at a nucleon–nucleon centre-of-mass energy of $\sqrt{s_{_\text {NN}}} = 5.02$ $\text {TeV}$ using the ATLAS detector at the Large Hadron Collider. Charged particles with absolute pseudorapidity less than 2.7 are reconstructed using the ATLAS pixel detector. The  collision centrality is characterised by the total transverse energy measured in the Pb-going direction of the forward calorimeter. The charged-particle pseudorapidity distributions are found to vary strongly with centrality, with an increasing asymmetry between the proton-going and Pb-going directions as the collisions become more central. Three different estimations of the number of nucleons participating in the  collision have been carried out using the Glauber model as well as two Glauber–Gribov inspired extensions to the Glauber model. Charged-particle multiplicities per participant pair are found to vary differently for these three models, highlighting the importance of including colour fluctuations in nucleon–nucleon collisions in the modelling of the initial state of  collisions.

## Introduction

Proton–nucleus () collisions at the Large Hadron Collider (LHC) [1] provide an opportunity to probe the physics of the initial state of ultra-relativistic heavy-ion (A $+$ A) collisions without the presence of thermalisation and collective evolution [2]. In particular,  measurements can provide insight into the effect of an extended nuclear target on the dynamics of soft and hard scattering processes and subsequent particle production. Historically, measurements of the average charged-particle multiplicity as a function of pseudorapidity, $\text {d}N_{\text {ch}}/\text {d}\eta$, where pseudorapidity is defined as $\eta =-\ln \tan (\theta /2)$ with $\theta$ the particle angle with respect to the beam direction, have yielded important insight into soft particle production dynamics in proton– and deuteron–nucleus () collisions [3–8] and provided essential tests of models of inclusive soft hadron production.

Additional information is obtained if measurements of the charged-particle multiplicities are presented as a function of centrality, an experimental quantity that characterises the  collision geometry. Previous measurements in  collisions at the Relativistic Heavy Ion Collider (RHIC) [9] have characterised the centrality using particle multiplicities at large pseudorapidity, either symmetric around mid-rapidity [10] or in the Au fragmentation direction [11]. These measurements have shown that the rapidity-integrated particle multiplicity in  collisions scales with the number of inelastically interacting, or “participating”, nucleons, $N_{\text {part}}$. This scaling behaviour has been interpreted as the result of coherent multiple soft interactions of the projectile nucleon in the target nucleus, and is known as the wounded–nucleon (WN) model [12]. The charged-particle multiplicity distributions as a function of pseudorapidity measured in central  collisions are asymmetric and peaked in the Au-going direction [7]. This observation has been explained using well-known phenomenology of soft hadron production [13].

There are alternative descriptions of the centrality dependence of the $\text {d}N_{\text {ch}}/\text {d}\eta$ distribution in  collisions at RHIC [14, 15] and  collisions at the LHC [15–17] based on parton saturation models. Measurements of the centrality dependence of $\text {d}N_{\text {ch}}/\text {d}\eta$ distributions in  collisions provide an essential test of soft hadron production mechanisms at the LHC. Such tests have become of greater importance given the observation of two-particle [18–21] and multi-particle [21–23] correlations in the final state of  collisions at the LHC. These correlations are currently interpreted as resulting from either initial-state saturation effects [15, 24, 25] or from the collective dynamics of the final state [26–30]. For either interpretation, information on the centrality dependence of $\text {d}N_{\text {ch}}/\text {d}\eta$ can provide important input for determining the mechanism responsible for these structures.

Recent measurements from the ALICE experiment [31] show behaviour in the centrality dependence of the charged-particle pseudorapidity distributions, which is qualitatively similar to that observed at RHIC. That analysis compared different methods for characterising centrality and suggested that the method used to define centrality may have a significant impact on the centrality dependence of the measured $\text {d}N_{\text {ch}}/\text {d}\eta$ distribution.

An important component of any centrality-dependent analysis is the geometric model used to relate experimental observables to the geometry of the nuclear collision. Glauber Monte Carlo models [32], which simulate the interactions of the incident nucleons using a semi-classical eikonal approximation, have been successfully applied to many different  measurements at RHIC and the LHC. A key parameter of such models is the inelastic nucleon–nucleon cross-section, which is taken to be 70 mb for this analysis [31]. However, the Glauber multiple-scattering approximation assumes that the nucleons remain on the mass shell between successive scatterings, and this assumption is badly broken in ultra-relativistic collisions. Corrections to the Glauber model [33], hereafter referred to as “Glauber–Gribov,” are needed to account for the off-shell propagation of the nucleons between collisions.

A particular implementation of the Glauber–Gribov approach is provided by the colour-fluctuation model [34–37]. That model accounts for event-to-event fluctuations in the configuration of the incoming proton that are assumed to be frozen over the timescale of a collision and that can change the effective cross-section with which the proton scatters off nucleons in the nucleus. These event-by-event fluctuations in the cross-section can be represented by a probability distribution $P(\sigma )$. The width of that distribution can be characterised by a parameter $\omega _{\sigma }$, which is the relative variance of the $\sigma$ distribution, $\omega _{\sigma } \equiv \langle \left( \sigma /\sigma _{\mathrm {tot}} - 1\right) ^2 \rangle$. The usual total cross-section, $\sigma _{\mathrm {tot}}$, is the event-averaged cross-section, or, equivalently, the first moment of the $P(\sigma )$ distribution, $\sigma _{\mathrm {tot}} = \int _0^{\infty }{d\sigma } P(\sigma )\, \sigma$. The parameter $\omega _{\sigma }$ can be measured using diffractive proton–proton scattering at high energy [35, 36]. First estimates of $\omega _{\sigma }$ at LHC energies [36] extrapolated to 5 TeV yielded $\omega _{\sigma } \sim 0.11$, while a more recent analysis suggested $\omega _{\sigma } \sim 0.2$ [37]. Because the cross-section fluctuations in the Glauber–Gribov colour-fluctuation (GGCF) model may have a significant impact on the interpretation of the results of this analysis, the geometry of  collisions has been evaluated using both the standard Glauber model and the GGCF model with $\omega _{\sigma } = 0.11$ and 0.2.

This paper presents measurements of the centrality dependence of $\text {d}N_{\text {ch}}/\text {d}\eta$ in  collisions at $\sqrt{s_{_\text {NN}}} = 5.02$ $\text {TeV}$ using $1~{\rm \mu} \mathrm{b}^{-1}$ of data recorded by the ATLAS experiment [38] in September 2012. Charged particles are detected in the ATLAS pixel detector and are reconstructed using a two-point tracklet algorithm similar to that used for the  multiplicity measurement [39]. Measurements of $\text {d}N_{\text {ch}}/\text {d}\eta$ are presented for several intervals in collision centrality characterised by the total transverse energy measured in the forward section of the ATLAS calorimeter on the Pb-going side of the detector. A standard Glauber model [32] and the GGCF model [36, 37] with $\omega _{\sigma } = 0.11$ and 0.2 are used to estimate $\langle N_{\text {part}} \rangle$ for each centrality interval, allowing a measurement of the $N_{\text {part}}$ dependence of the charged-particle multiplicity.

The paper is organised as follows. Section 2 describes the subdetectors of the ATLAS experiment relevant for this measurement. Section 3 describes the event selection. Section 4 describes the Monte Carlo simulations used to understand the performance and derive the corrections to the measured quantities. Section 5 describes the choice of centrality variable. Section 6 describes the measurement of the charged-particle multiplicity and Sect. 7 describes the estimation of the systematic uncertainties. Section 8 presents the results of the measurement, and the interpretation of the yields of charged particles per participant is discussed in Sect. 9. Section 10 concludes the paper. The estimation of the geometric parameters in each centrality interval for the Glauber and GGCF models is presented in detail in the “Appendix”.

## Experimental setup

The ATLAS detector is described in detail in Ref. [38]. The data selection and analysis presented in this paper is performed using the ATLAS inner detector (ID), calorimeters, minimum-bias trigger scintillators (MBTS), and the trigger system. The inner detector measures charged-particle tracks using a combination of silicon pixel detectors, silicon microstrip detectors (SCT), and a straw-tube transition-radiation tracker (TRT), all immersed in a 2 T axial magnetic field. The pixel detector is divided into “barrel” and “endcap” sections. For collisions occurring at the nominal interaction point,^1^ the barrel section of the pixel detector allows measurements of charged-particle tracks over $|\eta | < 2.2$. The endcap sections extend the detector coverage, spanning the pseudorapidity interval $1.6<|\eta |<2.7$. The SCT and TRT detectors cover $|\eta | < 2.5$ and $|\eta | < 2$, respectively, also through a combination of barrel and endcap sections.

The barrel section of the pixel detector consists of three layers of staves at radii of 50.5, 88.5 and 122.5 mm from the nominal beam axis, and extending $\pm 400.5$ mm from the centre of the detector in the *z* direction. The endcap consists of three disks placed symmetrically on each side of the interaction region at *z* locations of $\pm 493$, $\pm 578$ and $\pm 648$ mm from the centre of the detector. All pixel sensors in the pixel detector, in both the barrel and endcap regions, are identical and have a nominal size of $50\,{\upmu }\mathrm{m} \times 400\,{\upmu }\mathrm{m}$.

The MBTS detect charged particles in the range $2.1 < |\eta | < 3.9$ using two hodoscopes, each of which is subdivided into 16 counters positioned at $z=\pm 3.6$ m. The ATLAS calorimeters cover the full azimuth and the pseudorapidity range $|\eta |<4.9$ with the forward part (FCal) consisting of two modules positioned on either side of the interaction region and covering $\text{3.1 } < |\eta | < 4.9$. The FCal modules are composed of tungsten and copper absorbers with liquid argon as the active medium, which together provide ten interaction lengths of material.

The LHC delivered its first proton–nucleus collisions in a short  “pilot” run at $\sqrt{s_{_\text {NN}}} = 5.02$ $\text {TeV}$ in September 2012. During that run the LHC was configured with a clockwise 4 $\text {TeV}$ proton beam and an anti-clockwise 1.57 $\text {TeV}$ per-nucleon Pb beam that together produced collisions with a nucleon–nucleon centre-of-mass energy of $\sqrt{s_{_\text {NN}}} = 5.02$ $\text {TeV}$ and a longitudinal rapidity boost of 0.465 units with respect to the ATLAS laboratory frame. Following a common convention used for  measurements, the rapidity is taken to be positive in the direction of the proton beam, i.e. opposite to the usual ATLAS convention for $pp$ collisions. With this convention, the ATLAS laboratory frame rapidity *y* and the  centre-of-mass system rapidity $y_{\text {cm}}$ are related as $y_{\text {cm}} = y-0.465$.

## Event selection

Minimum-bias  collisions were selected by a trigger that required a signal in at least two MBTS counters. The  events selected for analysis are required to have at least one hit in each side of the MBTS, a difference between the times measured in the two MBTS hodoscopes of less than 10 ns, and a reconstructed collision vertex in longitudinal direction, $z_{\mathrm {vtx}}$, within 175 mm of the nominal centre of the ATLAS detector. Collision vertices are defined using charged-particle tracks reconstructed by an algorithm optimised for $pp$ minimum-bias measurements [40]. Reconstructed vertices are required to have at least two tracks with transverse momentum $p_\mathrm {T} > 0.4$ $\text {GeV}$. Events containing multiple  collisions are rare due to very low instantaneous luminosity during the pilot run and are further suppressed in the analysis by rejecting events with two collision vertices that are separated in *z* by more than 15 mm. Applying this selection reduces the fraction of events with multiple collisions from less than 0.07 to below 0.01 %.

To remove potentially significant contributions from electromagnetic and diffractive processes, the topology of the events was first analysed in a manner similar to that performed in a measurement of rapidity gap cross-sections in 7 $\text {TeV}$ proton–proton collisions [41]. The pseudorapidity coverage of the calorimeter, $-4.9 < \eta < 4.9$, is divided into $\Delta \eta = 0.2$ intervals, and each interval containing one or more clusters with $p_{\text {T}}$ greater than 0.2 $\text {GeV}$ is considered as occupied. To suppress the contributions from noise, clusters are considered only if they contained at least one cell with an energy at least four times the standard deviation of the cell noise distribution.

Then, the edge-gap on the Pb-going side of the detector is calculated as the distance in pseudorapidity between the detector edge $\eta =-4.9$ and the nearest occupied interval. Events with edge-gaps larger than two units of pseudorapidity typically result from electromagnetic or diffractive excitation of the proton and are removed from the analysis. The effect of this selection is identical to the requirement of a cluster with transverse energy $E_{\text {T}} >0.2$ $\text {GeV}$ to be present in the region $\eta < -2.9$. No requirement is imposed on edge-gaps on the proton-going side. The gap requirement removes, with good efficiency, a sample of events which are not naturally described in a Glauber picture of  collisions. This requirement removes a further fraction $f_{\text {gap}} = 1$ % of the events passing the vertex and MBTS cuts, yielding a total of 2.1 million events for this analysis. The result of this event selection is to isolate a fiducial class of  events, defined as inelastic  events that have a suppressed contribution from diffractive proton excitation events.

## Monte Carlo simulation

The response of the ATLAS detector and the performance of the charged-particle reconstruction algorithms are evaluated using one million minimum-bias 5.02 $\text {TeV}$ Monte Carlo (MC)  events, produced by version 1.38b of the Hijing event generator [42] with diffractive processes disabled. The four-momentum of each generated particle is longitudinally boosted by a rapidity of 0.465 to match the beam conditions in the data. The detector response to these events is fully simulated using Geant4 [43, 44]. The resulting events are digitised using conditions appropriate for the pilot  run and fully reconstructed using the same algorithms that are applied to the experimental data. This MC sample is primarily used to evaluate the efficiency of the ATLAS detector for the charged-particle measurements.

The detector response and event selection efficiencies for peripheral and diffractive  events have properties similar to those for inelastic or diffractive $pp$ collisions, respectively. To evaluate these responses and efficiencies, the $pp$ samples are generated at $\sqrt{s} = 5.02$ $\text {TeV}$ with particle kinematics boosted to match the  beam conditions. Separate samples of minimum-bias, single-diffractive, and double-diffractive $pp$ collisions with one million events each are produced using both Pythia6 [45] (version 6.425, AMBT2 parameter set (tune) [46], CTEQ6L1 PDF [47]) and Pythia8 [48] (version 8.150, 4C tune [49], MSTW2008LO PDF [50]), and simulated, digitised and reconstructed in the same manner as the  events. These six samples are primarily used for the Glauber model analysis described in the “Appendix”.

## Centrality selection

**Fig. 1:**
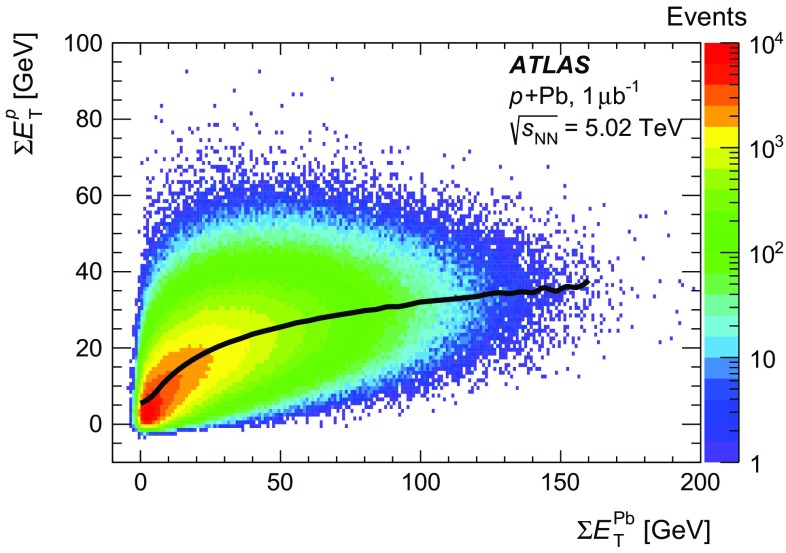
Distribution of proton-going ($\sum \!E_{\text {T}}^{p}$) versus Pb-going () total transverse energy in the forward calorimeter for  collisions included in this analysis. The *curve* shows the average $\sum \!E_{\text {T}}^{p}$ as a function of

For  collisions, the ATLAS experiment uses the total transverse energy, $\sum \!{E_\mathrm {T}}$, measured in the two forward calorimeter sections to characterise the collision centrality [51]. However, the intrinsic asymmetry of the  collisions and the rapidity shift of the centre-of-mass causes an asymmetry in the soft particle production measured on the two sides of the calorimeter. Figure 1 shows the correlation between the summed transverse energies measured in the proton-going ($3.1 < \eta < 4.9$) and Pb-going ($-4.9 < \eta < -3.1$) directions, $\sum \!E_{\text {T}}^{p}$ and , respectively. The transverse energies are evaluated at an energy scale calibrated for electromagnetic showers and have not been corrected for hadronic response.

**Fig. 2:**
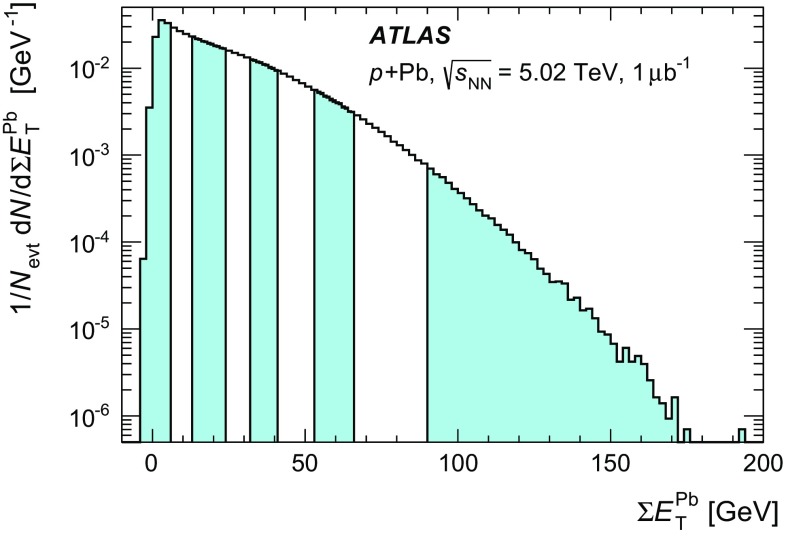
Distribution of the Pb-going total transverse energy in the forward calorimeter  values for events satisfying all analysis cuts including the Pb-going rapidity gap exclusion. The alternating *shaded* and *unshaded bands* indicate centrality intervals, from *right* (*central*) to *left* (*peripheral*), 0–1, 1–5, 5–10, 10–20, 20–30, 30–40, 40–60, 60–90 % and the interval 90–100 % that is not used in this analysis

Figure 1 shows that the mean $\sum \!E_{\text {T}}^{p}$ rapidly flattens with increasing  for  $\text {GeV}$, indicating that $\sum \!E_{\text {T}}^{p}$ is less sensitive than  to the increased particle production expected to result from multiple interactions of the proton in the target nucleus in central collisions. Thus,  alone, rather than , is chosen as the primary quantity used to characterise  collision centrality for the measurement presented in this paper. However, we describe alternate choices of the centrality-defining region below and evaluate the sensitivity of the measurement to this definition.

The distribution of  for events passing the  analysis selection is shown in Fig. 2. The following centrality intervals are defined in terms of percentiles of the  distribution: 0–1, 1–5, 5–10, 10–20, 20–30, 30–40, 40–60, and 60–90 %. The  ranges corresponding to these centrality intervals are indicated by the alternating filled and unfilled regions in Fig. 2, with the 0–1 % interval, containing the most central collisions, being rightmost. Since the composition of the events in the most peripheral 90–100 % interval is not well constrained, these events are excluded from the analysis. The nominal centrality intervals were defined after accounting for a 2 % inefficiency, as described in the “Appendix”, for the fiducial class of  events defined above to pass the applied event selection. Alternate intervals were also defined by varying this estimated inefficiency to 0 and 4 %, and is used as a systematic check on the results. While the inefficiency is confined to the 90–100 % interval, it influences the  ranges associated with each centrality interval. Potential hard scattering contributions to  have been evaluated in a separate analysis [52] by explicitly subtracting the contributions from reconstructed jets that fall partly or completely in the Pb-going FCal acceptance. That analysis showed negligible impact from hard scattering processes on the measured  distribution.

To test the sensitivity of the results to the choice of pseudorapidity interval used for the $\sum \!{E_\mathrm {T}}$ measurement, two alternative $\sum \!{E_\mathrm {T}}$ quantities are defined. The former, $\sum \!E_{\text {T}}^{\eta <-4}$, is defined as the total transverse energy in FCal cells with $\eta < -4.0$. The latter, $\sum \!E_{\text {T}}^{3.6<|\eta _{\text {cm}}|<4.4}$, is defined as the total transverse energy in the two intervals $4.0 < \eta < 4.9$ and $-4.0< \eta < -3.1$, an approximately symmetric interval when expressed in pseudorapidity in the centre of mass system $\eta _\mathrm{{cm}}$. The first of these alternatives is used to evaluate the potential auto-correlation between the measured charged-particle multiplicities and the centrality observable by increasing the rapidity gap between the two measurements. The second is used to evaluate the differences between an asymmetric (Pb-going) and symmetric (both sides) centrality observable. The effect of these alternative definitions is discussed in Sect. 8.

**Fig. 3:**
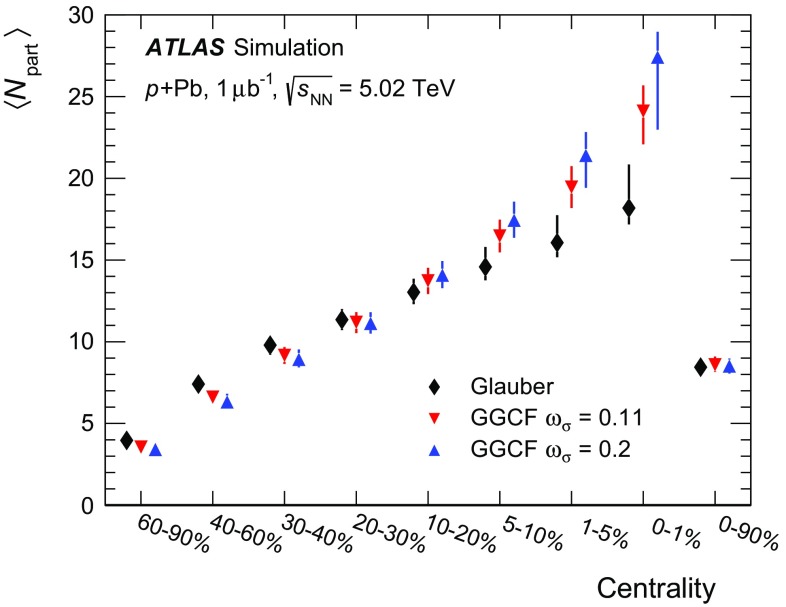
Mean value of the number of participating nucleons $\langle N_{\text {part}} \rangle$ for different centrality bins, resulting from fits to the measured  distribution using Glauber and Glauber–Gribov $N_{\text {part}}$ distributions. The *error bars* indicate asymmetric systematic uncertainties

The Glauber analysis [32] was applied to estimate $\langle N_{\text {part}} \rangle$ for each of the centrality intervals used in this analysis. A detailed description is given in the “Appendix”; only a brief summary of the method is given here. The PHOBOS MC program [53] was used to simulate the geometry of inelastic  collisions using both the standard Glauber and GGCF models. The resulting $N_{\text {part}}$ distributions are convolved with a model of the $N_{\text {part}}$-dependent  distributions, the parameters of which are obtained by fitting the measured  distribution. The average $N_{\text {part}}$ associated with each centrality interval is obtained with systematic uncertainties. The results are shown in Fig. 3 for the Glauber model and for the GGCF model with $\omega _{\sigma } = 0.11$ and 0.2.

## Measurement of charged-particle multiplicity

### Two-point tracklet and pixel track methods

The measurement of the charged-particle multiplicity is performed using only the pixel detector to maximise the efficiency for reconstructing charged particles with low transverse momenta. Two approaches are used in this analysis. The first is the two-point tracklet method commonly used in heavy-ion collision experiments [39, 54, 55]. Two variants of this method are implemented in this analysis to construct the $\text {d}N_{\text {ch}}/\text {d}\eta$ distribution and to estimate the systematic uncertainties, as described below. The second method uses “pixel tracks”, obtained by applying the full track reconstruction algorithm [56] only to the pixel detector. The pixel tracking is less efficient than the tracklet method as is justified later in the text, but provides measurements of the particle $p_{\text {T}}$. The $\text {d}N_{\text {ch}}/\text {d}\eta$ distribution measured using pixel tracks provides a cross-check on the primary measurement that is performed using the two-point tracklets.

In the two-point tracklet algorithm, the event vertex and clusters [57] on an inner pixel layer define a search region for clusters in the outer layers. The algorithm uses all clusters, except the clusters which have low energy deposits inconsistent with minimum-ionising particles originating from the primary vertex. The algorithm also rejects duplicate clusters resulting from the overlap of the pixel sensors or arising from a small set of pixels at the centre of the pixel modules that share readout channels [58]. Two clusters in a given layer of the pixel detector are considered as one if they have an angular separation $\sqrt{(\delta \phi )^2 + (\delta \eta )^2} < 0.02$, because they likely result from the passage of a single particle.

The pseudorapidity and azimuthal angle of the cluster in the innermost layer ($\eta ,\phi$) and their differences between the outer and inner layers ($\Delta \eta ,\Delta \phi$) are taken as the parameters of the reconstructed tracklet. The $\Delta \eta$ of a tracklet is largely determined by the multiple scattering of the incident particles in the material of the beam pipe and detector. This effect plays a less significant role in the $\Delta \phi$ of a tracklet, which is driven primarily by the bending of charged particles in the magnetic field, and hence one expects $\Delta \phi$ to be larger. The tracklet selection cuts are:

1 $$\begin{aligned} |\Delta \eta |< & {} 0.015, \quad |\Delta \phi | < 0.1, \end{aligned}$$

2 $$\begin{aligned} |\Delta \eta |< & {} |\Delta \phi |. \end{aligned}$$

Keeping tracklets with $|\Delta \phi | < 0.1$ corresponds to accepting particles with $p_{\text {T}} \gtrsim 0.1$ $\text {GeV}$. The selection in Eq. (2) accounts for the momentum dependence of charged-particle multiple scattering.

The Monte Carlo simulation for the $\text {d}N_{\text {ch}}/\text {d}\eta$ analysis is based on the Hijing event generator, which is described in Sect. 4. The Hijing event generator is known to not accurately reproduce the measured particle $p_{\text {T}}$ distributions. This is addressed by reweighting the Hijing $p_{\text {T}}$ distribution using the ratio of reconstructed spectra measured with the pixel track method in the data and in the MC simulation. The reweighting function is extrapolated below $p_{\text {T}} =0.1$ $\text {GeV}$ and applied to all generated particles and their decay products. This is done in intervals of centrality and pseudorapidity. Generator-level primary particles are defined as particles with a mean lifetime $\tau > 0.3\times 10^{-10}$ s either directly produced in  interactions or from subsequent decays of particles with a shorter lifetime. This definition is the same as used in previous measurements of charged-particle production in $pp$  [40] and  [59] collisions by ATLAS. All other particles are defined as secondaries. Tracklets are classified as primary or secondary depending on whether the associated generator-level charged particle is primary or secondary. Association between the tracklets and the generator-level particles is based on the Geant4 information about hits produced by these particles. Tracklets that are formed from the random association of hits produced by unrelated particles, or hits in the detector which are not matched to any generated particle are referred to as “fake” tracklets.

The contribution of fake tracklets is relatively difficult to model in the simulation, because of the *a priori* unknown contributions of multiple sources, such as noisy clusters or very low energy particles. To address this problem, the tracklet algorithm is used in two different implementations referred to as “Method 1 ” and “Method 2 ”. In Method 1, at most one tracklet is reconstructed for each cluster on the first pixel layer. If multiple clusters on the second pixel layer fall within the search region, the resulting tracklets are merged into a single tracklet. This approach reduces, but does not eliminate, the contribution of fake tracklets that are then accounted for using an MC-based correction. Method 2 reconstructs tracklets for all combinations of clusters in only two pixel layers, the innermost and the next-to-innermost detector layers. To account for the fake tracklets arising from random combinations of clusters, the same analysis is performed after inverting the *x* and *y* positions of all clusters on the second layer with respect to the primary vertex $(x-x_{\text {vtx}},y-y_{\text {vtx}})\rightarrow (-(x-x_{\text {vtx}}),-(y-y_{\text {vtx}}))$. The tracklet yield from this “flipped” analysis, $N_{\text {tr}} ^{\mathrm {fl}}$, is then subtracted from the original tracklet yield, $N_{\text {tr}} ^{\mathrm {ev}}$ to obtain an estimated yield of true tracklets $N_{\text {tr}}$,

3 $$\begin{aligned} N_{\text {tr}} (\eta ) = N_{\text {tr}} ^{\mathrm {ev}}(\eta ) - N_{\text {tr}} ^{\mathrm {fl}} (\eta ). \end{aligned}$$

**Fig. 4:**
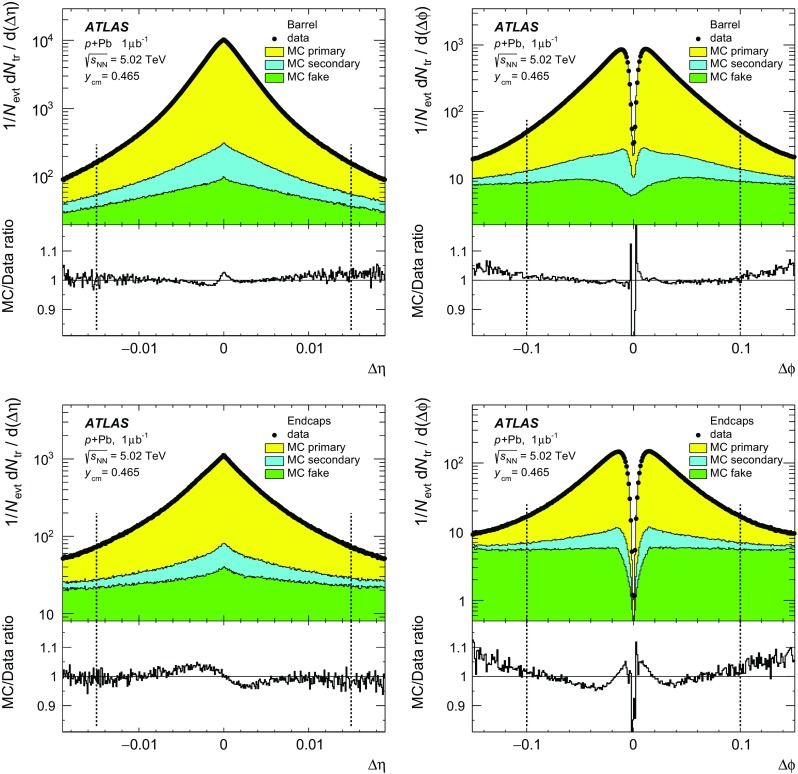
Stacked histograms for the differences between the hits of the tracklet in *outer* and *inner* detector layers in pseudorapidity $\Delta \eta$ (*left*) and in azimuth $\Delta \phi$ (*right*) for the tracklets reconstructed with Method 1 measured in the data (*points*) and simulation (*histograms*) in  collisions at $\sqrt{s_{_\text {NN}}}$ = 5.02 $\text {TeV}$ for barrel (*top*) and endcaps (*bottom*). Contributions from primary, secondary, and fake tracklets in the simulation are shown separately. The *lower panels* show the ratio of the simulation to the data

Distributions of $\Delta \eta$ and $\Delta \phi$ of reconstructed tracklets using Method 1 for data and simulated events are shown in Fig. 4 for the barrel (upper plots) and endcap (lower plots) parts of the pixel detector. The simulation results show the three contributions from primary, secondary and fake tracklets. The selection criteria specified by Eq. (1) are shown in Fig. 4 as vertical lines and applied in $\Delta \phi$ for $\Delta \eta$ plots and vice versa. Outside those lines, the contributions from secondary and fake tracklets are more difficult to take into account, especially in the endcap region. These contributions partially arise from low-$p_{\text {T}}$ particles on spiral trajectories and their description in the MC simulation is therefore very sensitive to the amount of detector material. The ratio between simulation and the data is also shown for each plot. These ratios are closer to unity in the barrel region than in the endcap region, where they deviate by up to 5 % except at very low $|\Delta \phi |$. At low $|\Delta \phi |$ corresponding to high $p_{\text {T}}$, the MC deviates from the data even after reweighing procedure based on pixel tracks. This is due to low resolution of pixel track at high $p_{\text {T}}$, however, the contribution of high-$p_{\text {T}}$ particles to $\text {d}N_{\text {ch}}/\text {d}\eta$ is negligible.

**Fig. 5:**
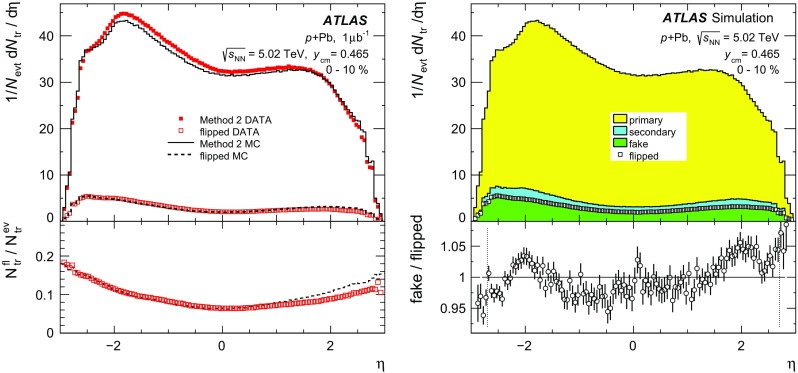
$\eta$-distribution of the number of tracklets reconstructed with Method 2. *Left top panel* comparison of the simulation (*lines*) to the data (*markers*). The results of the flipped reconstruction are shown with *open markers* for data and *dashed line* for simulation. *Right top panel* the simulated result for three contributions: primary, secondary and fake tracklets. *Square markers* show the result of simulation obtained with flipped reconstruction events. *Lower panels* on the *left* are the ratios of flipped ($N_{\text {tr}} ^{\mathrm {fl}}$) to direct ($N_{\text {tr}} ^{\mathrm {ev}}$) distribution in the data (*markers*) and in the simulation (*dashed line*); on the *right* is the ratio of the number of fake tracklets to the number of flipped tracklets

The top left panel of Fig. 5 shows the pseudorapidity distribution of tracklets reconstructed with Method 2 and satisfying the criteria of Eqs. (1) and (2) in the 0–10 % centrality interval for data (markers) and for the simulation (lines). The results of flipped reconstruction are also shown in the plot. The direct and flipped distributions are each similar between data and MC simulation but not identical, reflecting the fact that Hijing does not reproduce the data in detail. However, the lower panel of Fig. 5 shows that the ratios of the distribution of the number of tracklets in the flipped and direct events are very similar between the data and the MC simulation. A breakdown of the MC simulation distribution into primary, secondary and fake tracklets contributions is shown in the top right panel of Fig. 5. The distribution of $N_{\text {tr}} ^{\mathrm {fl}}(\eta )$, plotted with open markers, closely follows the histogram of fake tracklets. The lower panel of the plot shows the ratio of fake tracklet distribution to the flipped distribution. This ratio is consistent with unity to within 5 % in the entire range of measured $\eta$. This agreement justifies the subtraction of the fake tracklet contribution according to Eq. (3) for Method 2.

Although the fake rate is largest in Method 2, the flipped method is used to estimate the rate directly from the data. In the 0–10 % centrality interval, the fake tracklet contribution estimated with this method amounts to 8 % of the yield at mid-pseudorapidity and up to 16 % at large pseudorapidity. In the same centrality interval, the fake tracklet contributions using Method 1 and the pixel track method are smaller, vary from 2 to 10 and 0.2 to 1.5 %, respectively, but are determined with MC. All three methods rely on the MC simulation to correct for the contribution of secondary particles.

### Extraction of the charged-particle distribution

The data analysis and corresponding corrections are performed in eight intervals of detector occupancy ($\mathcal {O}$) parameterised using the number of reconstructed clusters in the first pixel layer and chosen to correspond to the eight  centrality intervals, and in seven intervals of $z_{\mathrm {vtx}}$, each 50 mm wide. For each analysis method, a set of multiplicative correction factors is obtained from MC simulations according to

4 $$\begin{aligned} C(\mathcal {O}, z_{\mathrm {vtx}}, \eta ) \equiv \frac{N_{\mathrm {pr}}(\mathcal {O},z_{\mathrm {vtx}},\eta )}{N_{\mathrm {rec}}(\mathcal {O}, z_{\mathrm {vtx}}, \eta )}. \end{aligned}$$

Here, $N_{\mathrm {pr}}$ and $N_{\mathrm {rec}}$ represent the number of primary charged particles at the generator level and the number of tracks or tracklets at the reconstruction level, respectively. These correction factors account for several effects: inactive areas in the detector and reconstruction efficiency, contributions of residual fake and secondary particles, and losses due to track or tracklet selection cuts including particles with $p_{\text {T}}$ below 0.1 $\text {GeV}$. They are evaluated as a function of $\mathcal {O}$, $z_{\mathrm {vtx}}$, and $\eta$ both for the fiducial region, $p_{\text {T}} > 0.1$ $\text {GeV}$, and for full acceptance, $p_{\text {T}} > 0$ $\text {GeV}$. The results are presented in $\eta$-intervals of 0.1 unit width. Due to the excellent $\eta$-resolution of the tracklets, as seen from Fig. 4, migration of tracklets between neighbouring bins is negligible.

**Fig. 6:**
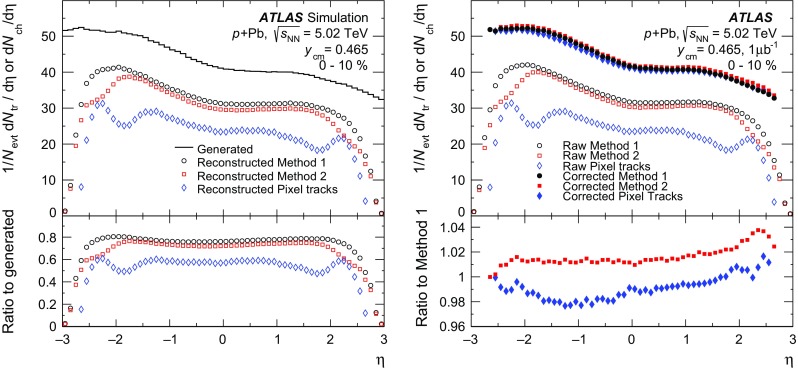
*Left top* distribution from the MC simulation for the generated number of primary charged particles per event ($\text {d}N_{\text {ch}}/\text {d}\eta$) shown with *line*, reconstructed number per event ($1/N_{\text {evt}} \text {d}N_{\text {tr}}/\text {d}\eta$) of tracklets from Method 1 shown with *circles*, tracklets from Method 2 after flipped event subtraction shown with *squares*, and pixel tracks shown with diamonds. *Left bottom* the ratio of reconstructed to generated tracklets and pixel tracks. *Right top*: *open markers* represent the same $1/N_{\text {evt}} \text {d}N_{\text {tr}}/\text {d}\eta$ distributions as in the *left panel*, reconstructed in the data. *Filled markers of the same shape* represent corrected distributions corresponding to $\text {d}N_{\text {ch}}/\text {d}\eta$. *Right bottom* the ratio of corrected distributions of Method 2 and pixel tracks to Method 1

The fully corrected, per-event charged-particle pseudorapidity distributions are calculated according to

5 $$\begin{aligned} \frac{\mathrm {d}N_{\text {ch}}}{\mathrm {d}\eta } = \frac{1}{\Delta \eta } \frac{\sum \Delta N_{\text {tr}} (\mathcal {O}, z_{\mathrm {vtx}}, \eta ) C(\mathcal {O}, z_{\mathrm {vtx}}, \eta )}{\sum N_{\text {evt}}(z_{\mathrm {vtx}})}, \end{aligned}$$

where $\Delta N_{\text {tr}}$ indicates either the number of reconstructed pixel tracks or two-point tracklets, $N_{\text {evt}}(z_{\mathrm {vtx}})$ is the number of analysed events in the intervals of the primary vertex along the *z* direction, and the sum in Eq. (5) runs over primary vertex intervals. The number of primary vertex intervals varies from seven for $|\eta |<2.2$ for two-point tracklets and $|\eta |<2$ for pixel tracks to two at the edges of the measured pseudorapidity range of $|\eta |<2.7$ for two-point tracklets and $|\eta |<2.5$ for pixel tracks respectively. The primary vertex intervals used in the analysis are chosen such that $C(\mathcal {O}, z_{\mathrm {vtx}}, \eta )$ changes by less than 20 % between any pair of adjacent $z_{\mathrm {vtx}}$ intervals.

**Table 1:** Summary of the various sources of systematic uncertainty and their estimated impact on the $\text {d}N_{\text {ch}}/\text {d}\eta$ measurement in central (0–1 %) and peripheral (60–90 %)  collisions

Source	60–90 % centrality		0–1 % centrality	
	Barrel (%)	Endcap (%)	Barrel (%)	Endcap (%)
Inactive modules		1.7		1.7
Extra material	0.5	3.0	0.5	3.0
Tracklet selection	0.5	1.5	0.5	1.5
$p_{\text {T}}$ reweighting	0.5	0.5	0.5	3.0
Particles with $p_{\text {T}} \le 0.1$ $\text {GeV}$	1.0	2.5	1.0	2.0
Particle composition		1.0		1.0
Contribution of fake tracklets	1.5	2.0	1.5	2.5
Event selection efficiency	5.0	6.0	0.5	0.5
Total	5.7	7.9	2.9	5.9

Figure 6 shows the effect of the applied correction for all three methods. The left panels shows the MC simulation results based on Hijing. The distribution of generated primary charged particles is shown by a solid line and the distributions of reconstructed tracks and tracklets are indicated by markers in the upper left panel. The lower left panel shows the ratio of reconstructed distributions to the generated distribution. Among the three methods, the corrections for Method 1 are the smallest, while the pixel track method requires the largest corrections. The structure of the measured distribution for the pixel track method around $\eta =\pm 2$ is related to the transition between the barrel and endcap regions of the detector. The open markers in the right panel of Fig. 6 show the reconstructed distribution from the data and the filled markers are the corresponding distribution for the three methods after applying corrections derived from the simulation. The lower panel shows the ratio of the results obtained from Method 2 and the pixel track method to that obtained using Method 1. The three methods agree within 2 % in the barrel region of the detector and within 3 % in the endcap region. This agreement demonstrates that the rejection of fake track or tracklets and the correction procedure are well understood. For this paper, Method 1 is chosen as the default result for $\text {d}N_{\text {ch}}/\text {d}\eta$, Method 2 is used when evaluating systematic uncertainties, and the pixel track method is used primarily as a consistency test, as discussed in detail below.

## Systematic uncertainties

The systematic uncertainties on the $\text {d}N_{\text {ch}}/\text {d}\eta$ measurement arise from three main sources: inaccuracies in the simulated detector geometry, sensitivity to selection criteria used in the analysis including the residual contributions of fake tracklets and secondary particles, and differences between the generated particles used in the simulation and the data. To determine the systematic uncertainties, the analysis is repeated in full for different variations of parameters or methods and the results are compared to the standard Method 1 results. A summary of the results are presented in Table  1.

The uncertainty due to the simulated detector geometry arises primarily from the details of the pixel detector acceptance and efficiency. The locations of the inactive pixel modules are matched between the data and simulation. Areas smaller than a single module that are found to have intermittent inefficiencies are estimated to contribute less than 1.7 % uncertainty to the final result. This uncertainty has no centrality dependence, and is approximately independent of pseudorapidity.

**Fig. 7:**
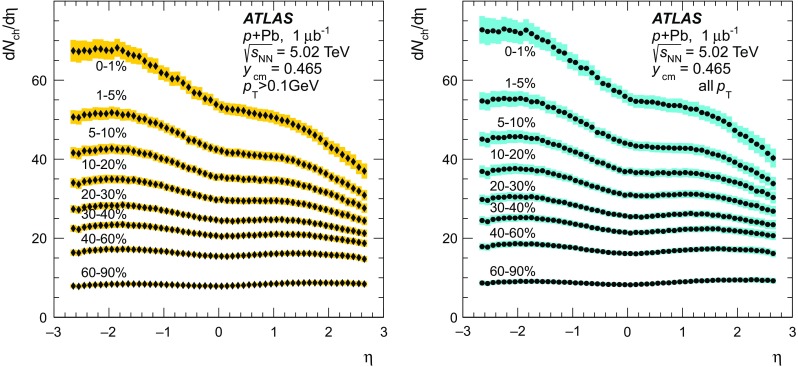
Charged-particle pseudorapidity distribution measured in several centrality intervals. *Left* $\text {d}N_{\text {ch}}/\text {d}\eta$ for charged particles with $p_{\text {T}} >0.1$ $\text {GeV}$. *Right* $\text {d}N_{\text {ch}}/\text {d}\eta$ for charged particles with $p_{\text {T}} >0$ $\text {GeV}$. Statistical uncertainties, shown with *vertical bars*, are typically smaller than the marker size. *Shaded bands* indicate systematic uncertainties on the measurements

The amount of inactive detector material in the tracking system is known with a precision of 5 % in the central region and up to 15 % in the forward region. In order to study the effect on the tracking efficiency, samples generated with increased material budget are used. The net effect on the final result is found to be 0.5–3 % independent of centrality.

Uncertainties due to tracklet selection cuts are evaluated by independently varying the cuts on $|\Delta \eta |$ and $|\Delta \phi |$ up and down by 40 %. The effect of these variations is less than 1 %, except at large values of $|\eta |$ where it is 1.5 %, and has only a weak centrality dependence.

The systematic uncertainty due to applying the $p_{\text {T}}$ reweighting procedure to the generated particles is taken from the difference in $\text {d}N_{\text {ch}}/\text {d}\eta$ between applying and not applying the reweighting procedure. The uncertainty is less than 0.5 % for $|\eta |<1.5$ and grows to 3.0 % towards the edges of the $\eta$ acceptance. The uncertainty has a centrality dependence because the $p_{\text {T}}$ distributions in central and peripheral collisions are different.

Tracklets are reconstructed using Method 1 for particles with $p_{\text {T}} >0.1$ $\text {GeV}$. The unmeasured region of the spectrum contributes approximately 6 % to the final $\text {d}N_{\text {ch}}/\text {d}\eta$ distribution. The systematic uncertainty on the number of particles with $p_{\text {T}} \le 0.1$ $\text {GeV}$ is partially included in the variation of the tracklet $\Delta \phi$ selection criteria. An additional uncertainty is evaluated by varying the shape of the spectra below 0.1 $\text {GeV}$. This uncertainty is estimated to be as much as 2.5 % at large values of $|\eta |$ and has a weak centrality dependence.

To test the sensitivity to the particle composition in Hijing, the fraction of pions, kaons and protons in Hijing are varied within a range based on measured differences in particle composition between $pp$ and  collisions [60, 61]. The resulting changes in $\text {d}N_{\text {ch}}/\text {d}\eta$ are found to be less than 1 % for all centrality intervals.

Systematic uncertainties due to the fake tracklets are estimated by comparing the results of the two tracklet methods. The differences in the most central collisions are found to vary with pseudorapidity from 1.5 % in the barrel region to about 2.5 % at the ends of the measured pseudorapidity range.

The uncertainty associated with the event selection efficiency for the fiducial class of  events is evaluated by defining new  centrality ranges after accounting for an increase (decrease) in the efficiency by 2 % and repeating the full analysis. This resulting change of the $\text {d}N_{\text {ch}}/\text {d}\eta$ distribution is less than 0.5 % in central collisions; it increases to 6 % in peripheral collisions.

The uncertainties from each source were evaluated separately in each centrality and pseudorapidity to allow for their partial or complete cancellation in the ratios of $\text {d}N_{\text {ch}}/\text {d}\eta$ distributions. The impact in different regions of pseudorapidity and centrality are shown in different columns of Table 1. Uncertainties coming from different sources and listed in the same column are treated as independent. The resulting total systematic uncertainty shown in the lower line of the table is the sum in quadrature of the individual contributions.

## Results

Figure 7 presents the charged-particle pseudorapidity distribution $\text {d}N_{\text {ch}}/\text {d}\eta$ for  collisions at $\sqrt{s_{_\text {NN}}}$ =5.02 $\text {TeV}$ in the pseudorapidity interval $|\eta |<2.7$ for several centrality intervals. The left panel shows the $\text {d}N_{\text {ch}}/\text {d}\eta$ distribution measured in the fiducial acceptance of the ATLAS detector, detecting particles with $p_{\text {T}} >0.1$ $\text {GeV}$. The results for the $\text {d}N_{\text {ch}}/\text {d}\eta$ distribution with $p_{\text {T}} >0$ $\text {GeV}$ are shown in the right panel of Fig. 7. The charged-particle pseudorapidity distribution increases by typically 5 %, consistent with extrapolation of spectra measured in $pp$ collisions to zero $p_{\text {T}}$  [40]. At the edges of the measured pseudorapidity interval, it increases $\text {d}N_{\text {ch}}/\text {d}\eta$ by 11 %.

**Fig. 8:**
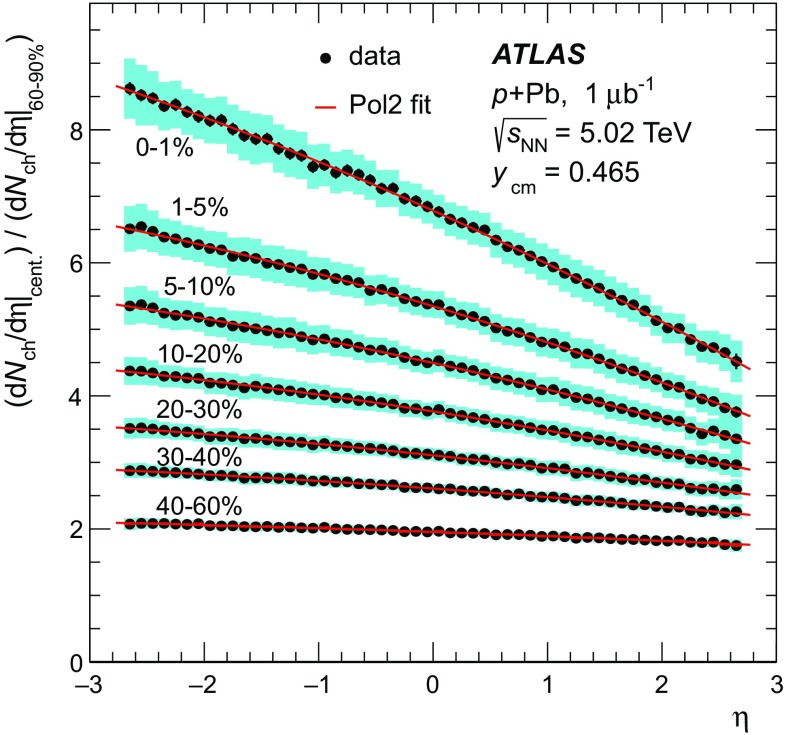
Ratios of $\text {d}N_{\text {ch}}/\text {d}\eta$ distributions measured in different centrality intervals to that in the *peripheral* (60–90 %) centrality interval. *Lines* show the results of second-order polynomial fits to the data points

In the most peripheral collisions with a centrality of 60–90 %, the $\text {d}N_{\text {ch}}/\text {d}\eta$ distribution has a doubly-peaked shape similar to that seen in $pp$ collisions [40, 62]. In collisions that are more central, the shape of $\text {d}N_{\text {ch}}/\text {d}\eta$ becomes progressively more asymmetric, with more particles produced in the Pb-going direction than in the proton-going direction. To investigate further the centrality evolution, the $\text {d}N_{\text {ch}}/\text {d}\eta$ distributions in each centrality interval are divided by the $\text {d}N_{\text {ch}}/\text {d}\eta$ distribution for the 60–90 % interval. The results are shown in Fig. 8, where the double-peak structure disappears in the ratios. The ratios are observed to grow nearly linearly with decreasing pseudorapidity, with a slope whose magnitude increases from peripheral to central collisions. In the 0–1 % centrality interval, the ratio changes by almost a factor of two over the measured $\eta$-range. The greatest increase in multiplicity between adjacent centrality intervals occurs between the 1–5 and 0–1 % intervals. Averaged over the $\eta$-interval of the measurement, the $\text {d}N_{\text {ch}}/\text {d}\eta$ distribution increases by more than 25 % between the 1–5 and 0–1 % intervals.

**Fig. 9:**
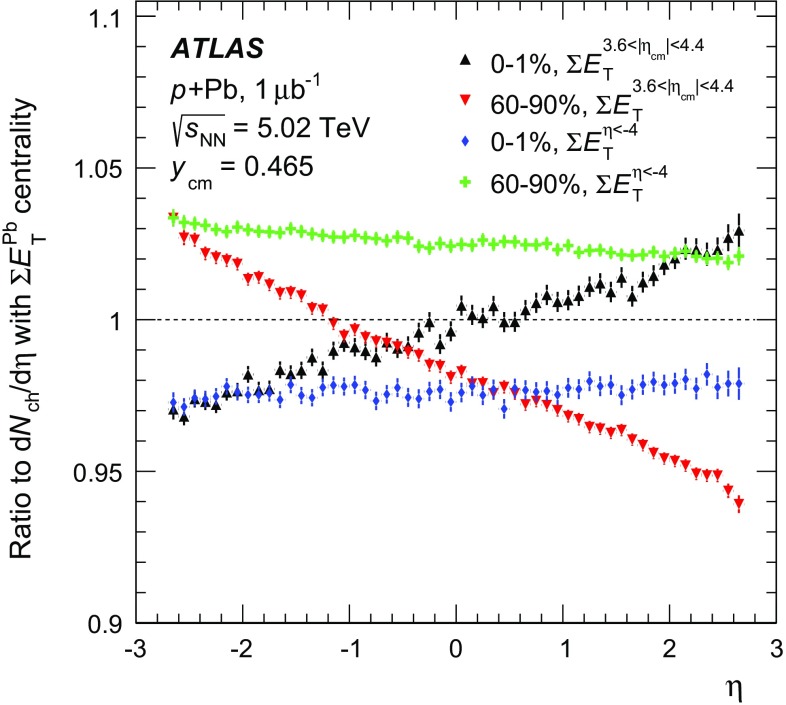
Ratios of $\text {d}N_{\text {ch}}/\text {d}\eta$ obtained using alternative centrality definitions to the nominal results presented in this paper as a function of $\eta$ for the 0–1 and 60–90 % centrality bins

In addition to the results presented in Figs. 7 and 8, the $\text {d}N_{\text {ch}}/\text {d}\eta$ measurement is repeated using the alternative definitions of the event centrality variables defined in Sect. 5. Figure 9 demonstrates the sensitivity of the measured $\text {d}N_{\text {ch}}/\text {d}\eta$ to the choice of centrality variable by showing the ratios of the $\text {d}N_{\text {ch}}/\text {d}\eta$ distributions in the most central and most peripheral intervals under the $\sum \!E_{\text {T}}^{\eta <-4}$ and $\sum \!E_{\text {T}}^{3.6<|\eta _{\text {cm}}|<4.4}$ centrality definitions to those obtained with the nominal  definition. Using the $\sum \!E_{\text {T}}^{\eta <-4}$ centrality definition, the $\text {d}N_{\text {ch}}/\text {d}\eta$ distributions change in an approximately $\eta$-independent fashion by $-3$ and $+3$ % for the 0–1 and 60–90 % intervals, respectively. The $\text {d}N_{\text {ch}}/\text {d}\eta$ distributions in the other centrality intervals change in a manner that effectively interpolates between these extremes. As a result, the increase in $\text {d}N_{\text {ch}}/\text {d}\eta$ between the most peripheral and most central collisions would be reduced by 6 % relative to the nominal measurement. Using the symmetric, $\sum \!E_{\text {T}}^{3.6<|\eta _{\text {cm}}|<4.4}$ centrality definition, the $\text {d}N_{\text {ch}}/\text {d}\eta$ distribution in each interval changes in an $\eta$-dependent way such that the ratio is consistent with a linear function of $\eta$. The change is at most 6 % at the ends of the $\eta$ range in the most central and most peripheral centrality intervals, and smaller elsewhere. Thus, for the symmetric centrality selection the ratios in Fig. 8 for the 0–1 % bin would increase by 9 % at $\eta = 2.7$, and decrease by 6 % at $\eta = -2.7$. Generally, the alternative centrality definitions considered in this analysis yield no qualitative and only modest quantitative changes in the centrality dependence of the $\text {d}N_{\text {ch}}/\text {d}\eta$ distributions. These variations should not be considered a systematic uncertainty on the $\text {d}N_{\text {ch}}/\text {d}\eta$ measurement but do indicate that the particular centrality method used in the analysis must be accounted for when interpreting the results of the measurement.

**Fig. 10:**
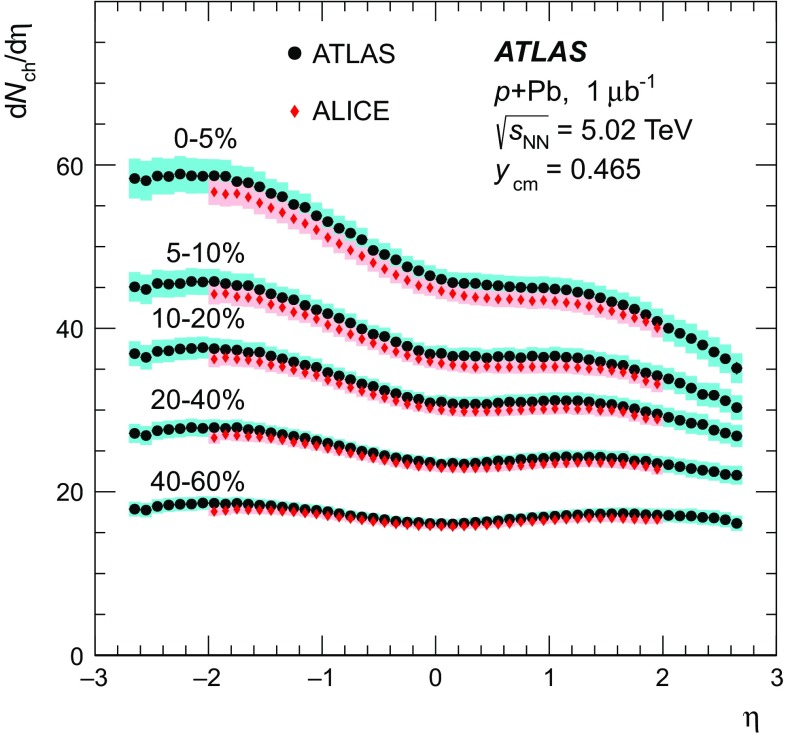
Charged-particle pseudorapidity distribution $\text {d}N_{\text {ch}}/\text {d}\eta$ measured in different centrality intervals compared to similar results from the ALICE experiment [31] using the “V0A” centrality selection. The ATLAS centrality intervals have been combined, where possible, to match the ALICE centrality selections

Figure 10 shows a comparison, where possible, of the measurements presented in this paper to results from the ALICE experiment [31] using a centrality definition that is based on the detector covering the pseudorapidity region $-5.1<\eta <-2.8$, similar to the -based selection used in this measurement. The ATLAS results for 0–1 and 1–5 % centrality intervals are combined to match the ALICE experiment result for 0–5 % interval. Similarly, the 20–30 and 30–40 % intervals are combined to match the ALICE experiment result for 20–40 % interval. The results from the two experiments are consistent with each other.

## Particle multiplicities per participant pair

**Fig. 11:**
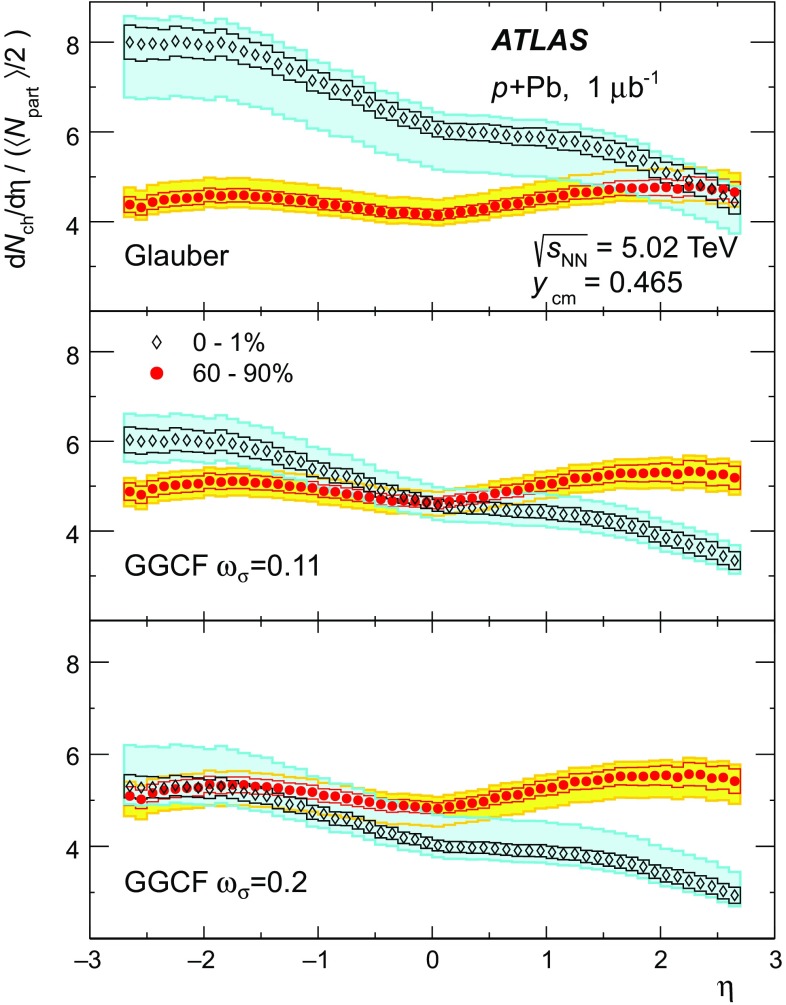
Charged-particle pseudorapidity distribution $\text {d}N_{\text {ch}}/\text {d}\eta$ per pair of participants as a function of $\eta$ for 0–1 and 60–90 % centrality intervals for the three models used to calculate $N_{\text {part}}$. The standard Glauber calculation is shown in the *top panel*, the GGCF model with $\omega _{\sigma }=0.11$ in the *middle* and $\omega _{\sigma }=0.2$ in the *lowest panel*. The *bands shown with thin lines* represent the systematic uncertainty of the $\text {d}N_{\text {ch}}/\text {d}\eta$ measurement, the *shaded bands* indicate the total systematic uncertainty including the uncertainty on $\langle N_{\text {part}} \rangle$. Statistical uncertainties, shown with *vertical bars*, are typically smaller than the marker size

A common way of representing the centrality dependence of particle yields in  and  collisions is by showing the yield per participant or per participant pair, $\langle N_{\text {part}} \rangle /2$, which is determined for each centrality interval and each geometrical model as shown in Fig. 3. Figure 11 shows $\text {d}N_{\text {ch}}/\text {d}\eta$ per participant pair for the most central and most peripheral intervals of centrality measured as a function of $\eta$ for three different models of the collisions geometry: the standard Glauber model and the GGCF model with $\omega _{\sigma } = 0.11$ and 0.2 in the top, middle and lower panels, respectively. The results for the most peripheral (60–90 %) centrality interval, shown with circles, are similar between all three panels. This is due to relatively small difference between the calculations of $\langle N_{\text {part}} \rangle$ for Glauber and GGCF models in this centrality interval. The shape of the distribution indicates more abundant particle production in the proton-going direction in comparison to the Pb-going. This can be explained by the higher energy of the proton compared to the energy of a single nucleon in the lead nucleus in the laboratory system. In the most central collisions (0–1 %), shown with diamond markers in all three panels, this trend is reversed. Conversely, the magnitude of $\text {d}N_{\text {ch}}/\text {d}\eta$ per participant pair strongly depends on the geometric model used to calculate $\langle N_{\text {part}} \rangle$. The point at which the central and peripheral scaled distributions cross each other also depends on the choice of geometric model.

**Fig. 12:**
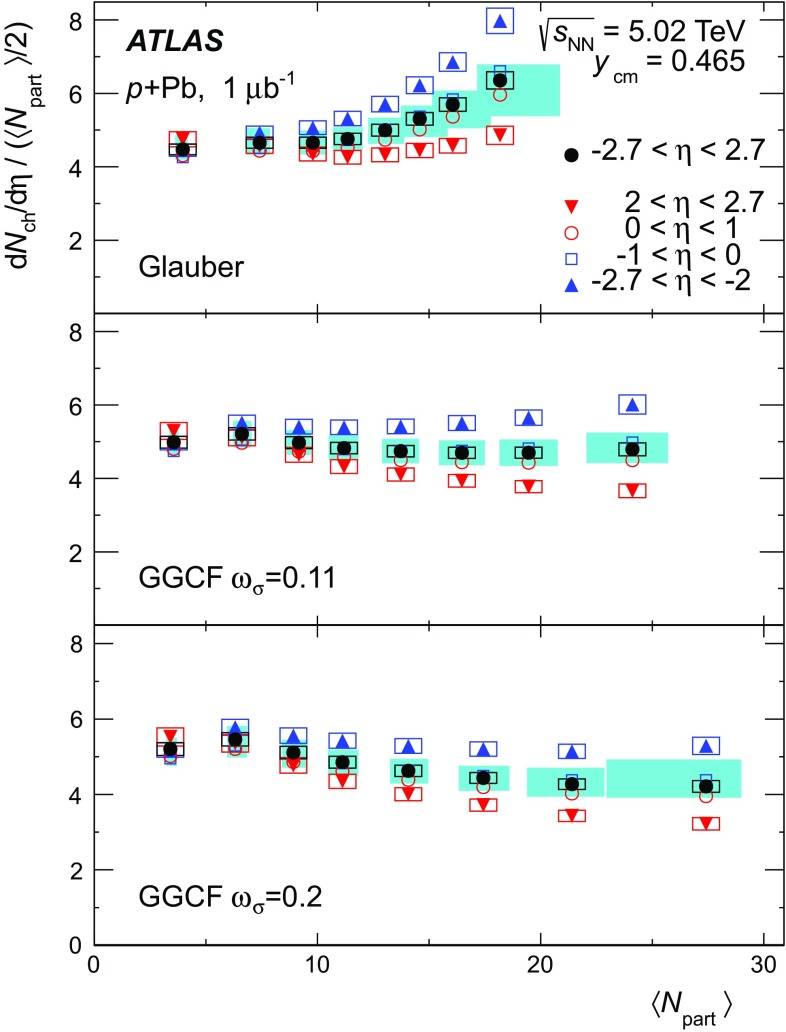
Charged-particle pseudorapidity distribution $\text {d}N_{\text {ch}}/\text {d}\eta$ per pair of participants as a function of $\langle N_{\text {part}} \rangle$ in several $\eta$-regions for the three models of the geometry: the standard Glauber model (*top panel*), the GGCF model with $\omega _{\sigma }=0.11$ (*middle panel*) and GGCF with $\omega _{\sigma }=0.2$ (*bottom panel*). The *open boxes* represent the systematic uncertainty of the $\text {d}N_{\text {ch}}/\text {d}\eta$ measurement only, and the width of the *box* is chosen for better visibility (they are not shown for $-1.0<\eta <0$ and $0<\eta <1$). The *shaded boxes* represent the total uncertainty (they are shown only on $-2.7<\eta <2.7$ interval for visibility) which is dominated by the uncertainty of the $\langle N_{\text {part}} \rangle$ given in Table 4 and Fig. 3. This uncertainty is asymmetric due to the asymmetric uncertainties on $\langle N_{\text {part}} \rangle$. The statistical uncertainties are smaller than the marker size for all points

Figure 12 shows the $\text {d}N_{\text {ch}}/\text {d}\eta$ distribution per participant pair as a function of $\langle N_{\text {part}} \rangle$ for the three different models of the collisions geometry. Since the charged-particle yields have significant pseudorapidity dependence, $\text {d}N_{\text {ch}}/\text {d}\eta /(\langle N_{\text {part}} \rangle /2)$ is presented in five $\eta$ intervals including the full pseudorapidity interval, $-2.7<\eta <2.7$. In the region $0<\eta <1$, the $\text {d}N_{\text {ch}}/\text {d}\eta$ distribution is consistent with an empirical fit to inelastic $pp$ data that suggest $\text {d}N_{\text {ch}}/\text {d}\eta$ increases with centre-of-mass energy, $\sqrt{s}$, as $\left( \propto s^{0.10}\right)$ [16].

The $\text {d}N_{\text {ch}}/\text {d}\eta$/($\langle N_{\text {part}} \rangle$/2) values from the standard Glauber model are approximately constant up to $\langle N_{\text {part}} \rangle \approx 10$ and then increase for larger $\langle N_{\text {part}} \rangle$. This trend is absent in the GGCF model with $\omega _{\sigma }=0.11$, which shows a relatively constant behaviour for the integrated yield divided by the number of participant pairs. The $\text {d}N_{\text {ch}}/\text {d}\eta /(\langle N_{\text {part}} \rangle /2)$ values from the GGCF model with $\omega _{\sigma }=0.2$ show a slight decrease with $\langle N_{\text {part}} \rangle$ in all $\eta$ intervals.

The presence or absence of $\langle N_{\text {part}} \rangle$ scaling does not suggest a preference for one or another of the geometric models. However, this study emphasises that considering fluctuations of the nucleon–nucleon cross-section in the GGCF model may lead to significant changes in the $N_{\text {part}}$ scaling behaviour of the  $\text {d}N_{\text {ch}}/\text {d}\eta$ data and, thus, their interpretations.

## Conclusions

This paper presents a measurement of the centrality dependence of the charged-particle pseudorapidity distribution, $\text {d}N_{\text {ch}}/\text {d}\eta$, measured in approximately 1 $\upmu$b$^{-1}$ of  collisions at a nucleon–nucleon centre-of-mass energy of $\sqrt{s_{_\text {NN}}}$ = 5.02 $\text {TeV}$ collected by the ATLAS detector at the LHC. The fully corrected measurements are presented for the fiducial acceptance of the ATLAS detector ($p_{\text {T}} > 0.1$ $\text {GeV}$) and in the full acceptance ($p_{\text {T}} >0$ $\text {GeV}$). The $\text {d}N_{\text {ch}}/\text {d}\eta$ distributions are presented as a function of pseudorapidity over the range $-2.7<\eta <2.7$ and as a function of collision centrality for the 0–90 %  collisions. The centrality is characterised using the energy deposited in the forward calorimeter covering $-4.9 < \eta < -\text{3.1 }$ in the Pb-going direction.

The shape of $\text {d}N_{\text {ch}}/\text {d}\eta$ evolves gradually with centrality from an approximately symmetric shape in the most peripheral collisions to a highly asymmetric distribution in the most central collisions. The ratios of $\text {d}N_{\text {ch}}/\text {d}\eta$ measured in different centrality intervals to the $\text {d}N_{\text {ch}}/\text {d}\eta$ distribution in the most peripheral interval are approximately linear in $\eta$ with a slope that is strongly dependent on centrality. It is noteworthy that the greatest increase in charged-particle multiplicity between successive centrality bins occurs between the 1–5 and 0–1 % centrality bins.

The results are also interpreted using models of the underlying collision geometry. The average number of participants in each centrality interval, $\langle N_{\text {part}} \rangle$, is estimated using a standard Glauber model Monte Carlo simulation with a fixed nucleon–nucleon cross-section, as well as with two Glauber–Gribov colour fluctuation models which allow the nucleon–nucleon cross-section to fluctuate event-by-event. The $N_{\text {part}}$ dependence of $\text {d}N_{\text {ch}}/\text {d}\eta /(\langle N_{\text {part}} \rangle /2)$ is found to be sensitive to the modelling of the  collision geometry, especially in the most central collisions: while the standard Glauber modelling leads to a strong increase in the multiplicity per participant pair for collisions in the centrality range (0–30) % the GGCF model produces a much milder centrality dependence.

These results point to the importance of understanding not just the initial state of the nuclear wave function, but also the fluctuating nature of nucleon–nucleon collisions themselves.

## Figures and Tables

**Fig. 13:**
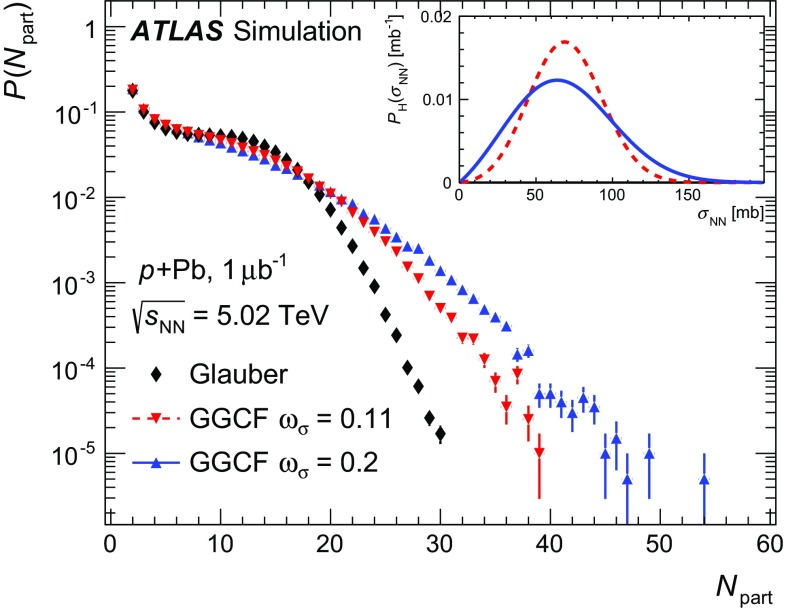
Glauber and GGCF $N_{\text {part}}$ distributions for 5.02 $\text {TeV}$  collisions obtained from one million simulated events each. The *inset* shows the GGCF $P_{\text {H}}(\sigma _{_\text {NN}})$ distributions for $\omega _{\sigma } = 0.11$ and 0.2

**Fig. 14:**
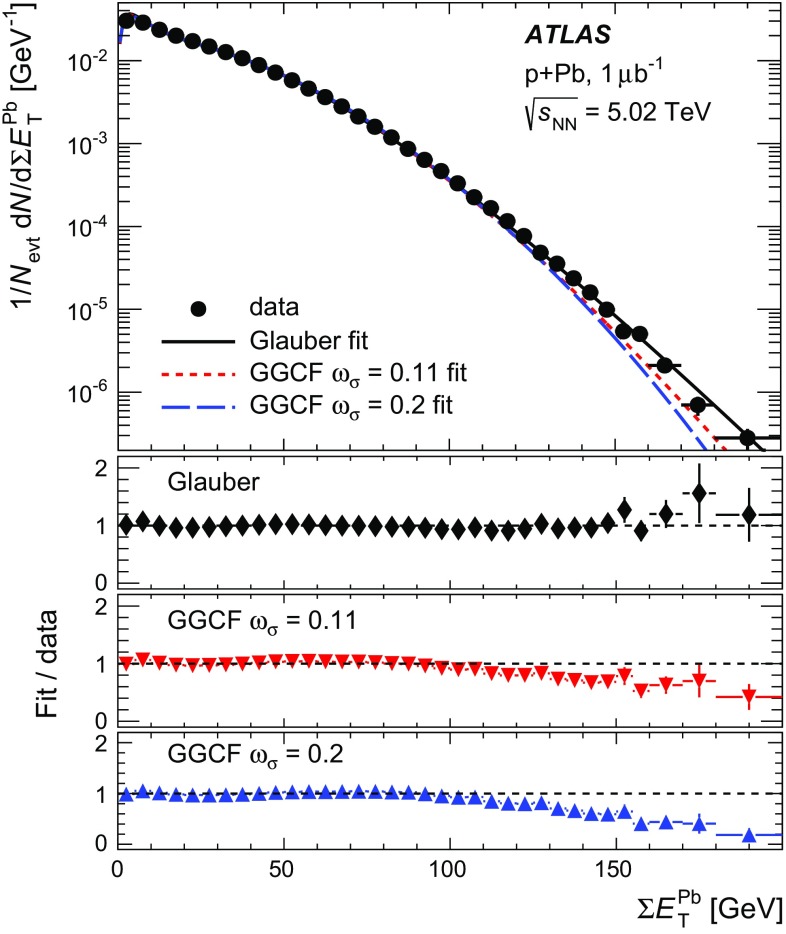
*Top panel* measured  distribution compared to Glauber (*solid*), GGCF with $\omega _{\sigma } = 0.2$ (*long dashed*), and GGCF with $\omega _{\sigma } = 0.11$ (*short dashed*) fits. *Lower panels* ratios of Glauber and GGCF fit distributions to the data distribution

## Appendix: Glauber model analysis

The PHOBOS Glauber MC program [53] is used to perform the standard Glauber model calculations used in this analysis. The Pb nucleon density is taken to be a Woods–Saxon distribution with radius and skin depth parameters, $R = 6.62$ fm and $a = 0.546$ fm [63], respectively. The nucleon–nucleon inelastic cross-section is taken to be 70 mb. The resulting probability distribution, $P(N_{\text {part}})$, of the number of participating nucleons $N_{\text {part}}$ – nucleons that undergo at least one hadronic scattering during the  collision – is shown in Fig. 13.

The GGCF model is implemented in a modified version of the PHOBOS MC program. Following Ref. [36], the probability distribution to find the nucleons in a configuration having a nucleon–nucleon scattering cross-section, $\sigma$, is taken to be

6 $$\begin{aligned} P ( \sigma ) = \rho \left( \frac{ \sigma }{ \sigma + \sigma _{\text {0}}} \right) \exp \left\{ - \frac{ (\sigma / \sigma _{\text {0}}- 1)^2 }{ \Omega ^2 } \right\} . \end{aligned}$$

Here, $\rho$ is a normalisation constant, $\Omega$ controls the width of the $P(\sigma )$ distribution, and $\sigma _{\text {0}}$ determines the configuration-averaged total cross-section $\sigma _{\text {tot}} \equiv \langle \sigma \rangle$. The inelastic cross-section, $\sigma _{_\text {NN}}$, is taken to be a constant fraction, $\lambda$, of the total cross-section [37] so the probability distribution of $\sigma _{_\text {NN}}$ is given by

7 $$\begin{aligned} P_{\text {H}}(\sigma _{_\text {NN}}) = \frac{1}{\lambda } P(\sigma _{_\text {NN}}/\lambda ). \end{aligned}$$

The values used in this analysis for $\Omega$, $\sigma _{\text {0}}$, $\sigma _{\text {tot}}$ and $\lambda$ corresponding to $\omega _{\sigma } = 0.11, 0.2$ are shown in Table 2. The first, earlier analysis yielding $\omega _{\sigma } = 0.11$ [36] assumed $\sigma _{\text {tot}} = 86$ mb, consistent with the Donnachie and Landshoff [64] parameterisation of $\sigma _{\text {tot}} \left( s\right)$. The second analysis yielding $\omega _{\sigma } = 0.2$ used an updated measurement of the $pp$ total cross-section at the LHC [65] to set $\sigma _{\text {tot}} = 94.8$ mb. However, modifying the parameters for the $\omega _{\sigma } = 0.11$ case to be consistent with this improved knowledge of $\sigma _{\text {tot}}$ produces a negligible change in the resulting $P(\sigma )$ distribution. The values for $\lambda$ are chosen to produce the above-quoted nucleon–nucleon inelastic cross-section of 70 mb. The GGCF $P_{\text {H}}(\sigma _{_\text {NN}})$ distributions are shown in the inset of Fig. 13, while the resulting $P(N_{\text {part}})$ distributions are shown in the main panel of the figure.

**Table 2:** Parameters used in the parameterisation of the GGCF $P(\sigma _\mathrm {tot})$ distribution

Parameter	$\omega _{\sigma } = 0.11$	$\omega _{\sigma } = 0.2$
$\Omega$	0.55	1.01
$\sigma _{\text {0}}$ (mb)	78.6	72.6
$\sigma _{\mathrm {tot}}$ (mb)	86	94.8
$\lambda$	0.82	0.74

To connect an experimental measurement of collision centrality such as  to the results of the Glauber or GGCF Monte Carlo simulation, a model for the $N_{\text {part}}$ dependence of the  distribution is required. The usual basis for models previously applied to  and  collisions is the WN model [12], which predicts that the average  increases proportionally to $N_{\text {part}}$ with the proportionality constant equal to one half the corresponding average FCal $\sum \!{E_\mathrm {T}}$ in $pp$ collisions.

Under the WN model, the  distribution for fixed $N_{\text {part}}$ would be obtained from a $N_{\text {part}}$-fold convolution of the corresponding distribution in $pp$ collisions. This convolution is straightforward if the $\sum \!{E_\mathrm {T}}$ distribution in $pp$ collisions is described by a gamma distribution [66]

8 $$\begin{aligned} \mathrm{gamma}\left( {\textstyle \sum } E_{\mathrm {T}}; k, \theta \right) = \frac{1}{\Gamma (k)}\frac{1}{\theta } \left( \frac{\sum \!{E_\mathrm {T}}}{\theta }\right) ^{k -1} e^{-\sum \!{E_\mathrm {T}}/\theta }, \end{aligned}$$

since gamma distributions have the property that an *N*-fold convolution of a gamma distribution with parameters *k* and $\theta$ yields another gamma distribution with the same $\theta$ and a modified *k* parameter, $k' = Nk$.

Attempts to fit the measured  distribution using pure WN-convolved gamma distributions and the Glauber $N_{\text {part}}$ distribution yield unphysical results for the nucleon–nucleon parameters, $k_0$ and $\theta _0$, when those parameters are free parameters of the fit. In particular, $k_0$ is less than unity, which implies a $\sum \!{E_\mathrm {T}}$ distribution that increases with decreasing $\sum \!{E_\mathrm {T}}$ faster than $e^{-\sum \!{E_\mathrm {T}}/\theta _0}$, and $\theta _0$ is unrealistically large. The resulting nucleon–nucleon $\sum \!{E_\mathrm {T}}$ distribution is also inconsistent with that measured in $pp$ collisions [67]. The poor behaviour of the WN model is primarily due to the difference in shape between the Glauber $N_{\text {part}}$ distribution and the measured  distribution. To improve the description of the measured  distribution, a generalisation of the WN model is implemented that parameterises the $N_{\text {part}}$ dependence of the *k* and $\theta$ parameters of the gamma distribution as

9 $$\begin{aligned} k\left( N_{\text {part}} \right)= & {} k_0 + k_1 \left( N_{\text {part}}- 2 \right) ,\nonumber \\ \theta \left( N_{\text {part}} \right)= & {} \theta _0 + \theta _1 \log {\left( N_{\text {part}}- 1 \right) }. \end{aligned}$$

For $k_1 = k_0/2$ and $\theta _1 = 0$, this model reduces to the WN model. The $\log \left( N_{\text {part}}- 1\right)$ term allows for a possible variation in the effective acceptance of the FCal due to an $N_{\text {part}}$-dependent backward shift in the  centre-of-mass system [68].

To limit the number of free parameters when fitting the  distribution, $k_0$ and $\theta _0$ are obtained by fitting the detector-level  distributions in Pythia6 and Pythia8 *pp* simulations. These simulations have been shown to give a reasonable description of the corresponding $pp$ collision data at $\sqrt{s} = 7$ $\text {TeV}$ [67] although they both slightly under-predict the average forward transverse energy. The contribution of electronic noise to the simulated distribution was determined by examining the  distribution in empty beam bunch crossings in data. In the fit, the gamma distributions were convolved with the effects of this noise before comparison with the data. The Pythia8 fit results, $k_0 = 1.40$ and $\theta _0 = 3.41$, are used for the default analysis. The Pythia6 fit results, $k_0 = 1.23$ and $\theta _0 = 2.68$, are used to evaluate systematic uncertainties.

The measured  distribution is fitted with a distribution produced by summing the $N_{\text {part}}$-dependent gamma distributions, after weighting them by $P(N_{\text {part}})$ and including an additional convolution to account for electronic noise. The model distribution is also re-weighted to properly describe the -dependent event selection efficiency in the data, which is estimated using the Pythia MC samples under the assumption that the  inefficiency for a given  is the same as that in $pp$ collisions. Results are shown in Fig. 14 for the Glauber model and the two GGCF models. The fits provide a good description of the  distribution for  $\text {GeV}$ for all three geometric models, although at higher  the Glauber fit describes the data better. The deviations of the GGCF fits from the data become significant near  $\text {GeV}$; the fraction of the total  distribution above this value is approximately 0.1 %.

**Table 3:** Optimal fit parameters and uncertainties obtained from fits to the measured  distribution. Uncertainties on the fit parameters are shown in parenthesis

Glauber model	$k_1$	$k_1/k_0$	$\theta _1$
Standard Glauber	0.425(2)	0.304(1)	$+1.32(1)$
GGCF $\omega _{\sigma } = 0.11$	0.901(3)	0.643(2)	$+0.074(4)$
GGCF $\omega _{\sigma } = 0.2$	1.139(3)	0.813(2)	$-0.209(2)$

**Table 4:** $\langle N_{\text {part}} \rangle$ values for centrality intervals used in this analysis together with asymmetric systematic uncertainties shown as absolute as well as relative uncertainties

Centrality (%)	Glauber	GGCF $\omega _{\sigma } = 0.11$	GGCF $\omega _{\sigma } = 0.2$
60–90	$4.0^{+0.2}_{-0.3} \left( ^{+5\%}_{-8\%}\right)$	$3.6^{+0.2}_{-0.2} \left( ^{+5\%}_{-5\%}\right)$	$3.4^{+0.3}_{-0.2} \left( ^{+8\%}_{-5\%}\right)$
40–60	$7.4^{+0.4}_{-0.6} \left( ^{+6\%}_{-8\%}\right)$	$6.6^{+0.4}_{-0.4} \left( ^{+6\%}_{-6\%}\right)$	$6.3^{+0.5}_{-0.3} \left( ^{+8\%}_{-5\%}\right)$
30–40	$9.8^{+0.6}_{-0.6} \left( ^{+6\%}_{-6\%}\right)$	$9.2^{+0.5}_{-0.5} \left( ^{+6\%}_{-6\%}\right)$	$8.9^{+0.6}_{-0.5} \left( ^{+7\%}_{-5\%}\right)$
20–30	$11.4^{+0.6}_{-0.6} \left( ^{+6\%}_{-6\%}\right)$	$11.2^{+0.6}_{-0.7} \left( ^{+6\%}_{-6\%}\right)$	$11.1^{+0.7}_{-0.6} \left( ^{+6\%}_{-6\%}\right)$
10–20	$13.0^{+0.8}_{-0.7} \left( ^{+6\%}_{-6\%}\right)$	$13.7^{+0.8}_{-0.8} \left( ^{+6\%}_{-7\%}\right)$	$14.1^{+0.9}_{-0.8} \left( ^{+6\%}_{-6\%}\right)$
5–10	$14.6^{+1.2}_{-0.8} \left( ^{+8\%}_{-6\%}\right)$	$16.5^{+1.0}_{-1.0} \left( ^{+6\%}_{-6\%}\right)$	$17.4^{+1.1}_{-1.1} \left( ^{+7\%}_{-6\%}\right)$
1–5	$16.1^{+1.7}_{-0.9} \left( ^{+11\%}_{-6\%}\right)$	$19.5^{+1.3}_{-1.3} \left( ^{+7\%}_{-7\%}\right)$	$21.4^{+1.4}_{-2.0} \left( ^{+7\%}_{-9\%}\right)$
0–1	$18.2^{+2.7}_{-1.0} \left( ^{+15\%}_{-5\%}\right)$	$24.1^{+1.6}_{-2.0} \left( ^{+7\%}_{-8\%}\right)$	$27.4^{+1.6}_{-4.0} \left( ^{+6\%}_{-16\%}\right)$
0–90	$8.4^{+0.5}_{-0.4} \left( ^{+6\%}_{-5\%}\right)$	$8.5^{+0.5}_{-0.5} \left( ^{+6\%}_{-5\%}\right)$	$8.6^{+0.5}_{-0.4} \left( ^{+6\%}_{-5\%}\right)$

The parameters $k_1$ and $\theta _1$ are obtained from fixing $k_0$ and $\theta _0$ and fitting the $\sum \!{E_\mathrm {T}}$ distribution to the data. They are presented in Table 3 along with the ratios $k_1/k_0$ for each of the geometric models. Pure WN behaviour would correspond to $k_1/k_0 = 0.5$ and $\theta _1 = 0$. The results indicate substantial deviations from WN behaviour for the Glauber and GGCF $\omega _{\sigma } = 0.2$ fits, while the GGCF $\omega _{\sigma } = 0.11$ fit yields both a $k_1/k_0$ that is close to 0.5 and a small $\theta _1$. The success of the above-described fitting procedure in describing the measured  distributions using parameterisations of the $N_{\text {part}}$ dependence of the  response with only two free parameters is due to the similarity of the shapes of the $P(N_{\text {part}})$ distribution and the measured  distributions. However, the pronounced knee in the Glauber $P(N_{\text {part}})$ distribution requires more non-linearity in the $N_{\text {part}}$ dependence of the  response. In contrast, the lack of such a feature in the GGCF $P(N_{\text {part}})$ distributions allows a simpler description of the measured  distribution.

The results of the fit procedure described above provide a data-driven estimate of the total  event-selection efficiency. For the default Pythia8-based results, the integral of the simulated  distribution is 2 % higher than that in the data. The deficit in the data is concentrated at low  values, consistent with losses due to event selection. However, a detailed analysis of the residual differences between the best fit and measured distributions indicates an excess of very low  events in the data, which varies from $\sim$ $0\,\%$ for the Glauber fit to 1.8 % for the GGCF fit with $\omega _{\sigma } = 0.2$. These may arise from residual diffractive or photo-nuclear collisions and are considered background. For the purpose of defining  centrality intervals, this background effectively increases the event-selection efficiency by adding events that are all in the 90–100 % centrality interval. For Pythia6, the  fits using the default model yield a total efficiency of 97 % and up to 1 % background. The alternative models for $k\left( N_{\text {part}} \right)$ and $\theta \left( N_{\text {part}} \right)$ yield a similar total efficiency of 98 % and a background rate as high as 2 %, a rate that is compatible with independent estimates of the rate for collisions involving diffractive excitation of the proton to pass the applied event selections. Based on these results, the total effective efficiency including background is then taken to be 98 % and the uncertainty is conservatively estimated to be 2 %.

The $\langle N_{\text {part}} \rangle$ values are obtained for each of the centrality intervals using the results of the fits to the  distributions. The $\langle N_{\text {part}} \rangle$ results along with the total systematic uncertainties, which are described below, are shown in Fig. 3 and listed in Table 4.

To obtain systematic errors on $N_{\text {part}}$ in each centrality interval, the maximum positive and negative fractional variation in $\langle N_{\text {part}} \rangle$ away from the default results is determined for different classes of variations, detailed below.

To evaluate the impact of the total event selection uncertainty, new centrality intervals are chosen assuming a total efficiency of 100 and 96 % and the complete analysis is repeated. To account for possible inaccuracies in the Pythia8-simulated $\mathrm {d}E_{\text {T}}/\mathrm {d}\eta$ in the region of the FCal acceptance, the analysis is repeated separately under $\pm 10\,\%$ re-scalings of Pythia8  values commensurate with the scale of the data–Pythia8 differences observed in Ref. [67]. Other variations for which the complete analysis is repeated are: (i) using the Pythia6 event generator to fix $k_0$ and $\theta _0$, (ii) alternative models for $k\left( N_{\text {part}} \right)$ and $\theta \left( N_{\text {part}} \right)$, (iii) $\pm 5$ mb changes in $\sigma _{_\text {NN}}$, and (iv) variations in the parameters of the nuclear density distribution. For the model uncertainty, two alternative parameterisations for $k\left( N_{\text {part}} \right)$ and $\theta \left( N_{\text {part}} \right)$ are used. One of these kept $\theta$ constant, $\theta \left( N_{\text {part}} \right) = \theta _0$ while allowing for a quadratic dependence of *k* on $N_{\text {part}}$. The other included both a quadratic term in $k\left( N_{\text {part}} \right)$ and the logarithmic term in $\theta \left( N_{\text {part}} \right)$ but fixed $k_1 = k_0/2$ to reduce the number of free parameters.

The resulting maximal variations are then summed in quadrature over the different classes separately for the positive and negative variations to produce the uncertainties listed in Table 4. The uncertainties for most centrality intervals are dominated by comparable contributions from the choice of model parameterisation, differences between the Pythia6 and Pythia8 $\sum \!{E_\mathrm {T}}$ fits, and uncertainties in $\sigma _{_\text {NN}}$. For the 40–60 and 60–90 % interval the uncertainty in event selection efficiency has a contribution to the $N_{\text {part}}$ systematic uncertainty that is similar in magnitude to these other three sources.

## Footnotes

1 ATLAS uses a right-handed coordinate system with its origin at the nominal interaction point (IP) in the centre of the detector and the *z*-axis along the beam pipe. The *x*-axis points from the IP to the centre of the LHC ring, and the *y*-axis points upward. Cylindrical coordinates $(r,\phi )$ are used in the transverse plane, $\phi$ being the azimuthal angle around the beam pipe. The pseudorapidity is defined in terms of the polar angle $\theta$ as $\eta =-\ln \tan (\theta /2)$.
